# Nucleolin mediates the internalization of rabbit hemorrhagic disease virus through clathrin-dependent endocytosis

**DOI:** 10.1371/journal.ppat.1007383

**Published:** 2018-10-19

**Authors:** Jie Zhu, Qiuhong Miao, Jingyu Tang, Xiaoxue Wang, Dandan Dong, Teng Liu, Ruibin Qi, Zhibiao Yang, Guangqing Liu

**Affiliations:** 1 Shanghai Veterinary Research Institute, Chinese Academy of Agricultural Sciences, Shanghai, P. R. China; 2 Laboratory of Virology, Wageningen University and Research, Wageningen, The Netherlands; 3 Shanghai Key Laboratory of Veterinary Biotechnology, Shanghai Jiao Tong University, Shanghai, P. R. China; Division of Clinical Research, UNITED STATES

## Abstract

Rabbit hemorrhagic disease virus (RHDV) is an important member of the *Caliciviridae* family and a highly lethal pathogen in rabbits. Although the cell receptor of RHDV has been identified, the mechanism underlying RHDV internalization remains unknown. In this study, the entry and post-internalization of RHDV into host cells were investigated using several biochemical inhibitors and RNA interference. Our data demonstrate that rabbit nucleolin (NCL) plays a key role in RHDV internalization. Further study revealed that NCL specifically interacts with the RHDV capsid protein (VP60) through its N-terminal residues (aa 285–318), and the exact position of the VP60 protein for the interaction with NCL is located in a highly conserved region (^472^Asp-Val-Asn^474^; DVN motif). Following competitive blocking of the interaction between NCL and VP60 with an artificial DVN peptide (RRTGDVNAAAGSTNGTQ), the internalization efficiency of the virus was markedly reduced. Moreover, NCL also interacts with the C-terminal residues of clathrin light chain A, which is an important component in clathrin-dependent endocytosis. In addition, the results of animal experiments also demonstrated that artificial DVN peptides protected most rabbits from RHDV infection. These findings demonstrate that NCL is involved in RHDV internalization through clathrin-dependent endocytosis.

## Author summary

Rabbit hemorrhagic disease virus (RHDV) is the causative agent of a highly contagious and lethal disease in rabbits. Since, at present, there is no robust cell culture system for the propagation of the virus, the molecular mechanism of RHDV internalization remains poorly understood. Here, we demonstrated that rabbit nucleolin (NCL) efficiently mediates RHDV internalization by interacting with the RHDV capsid protein (VP60). The exact function domains of the interaction between NCL and VP60 were also determined. Notably, these functional domains are highly conserved in all RHDV genotypes. Further study results revealed that NCL was involved in clathrin-dependent endocytosis through interactions with the C-terminal residues of clathrin light chain A. In addition, the artificial peptide (RRTGDVNAAAGSTNGTQ; DVN peptide) based on the functional domain, which is responsible for RHDV VP60 binding to NCL, is able to inhibit RHDV infection, indicating that the DVN peptide might be a candidate target for the design of antiviral drugs against RHDV infection.

## Introduction

Rabbit hemorrhagic disease virus (RHDV) is a non-enveloped, single-stranded, positive sense RNA virus, belonging to the *Caliciviridae* family [1], and it is the causative agent of a highly contagious and lethal disease in rabbits which is strongly associated with liver degeneration and diffuse hemorrhage [2,3]. RHDV was first isolated in China in 1984 [4] and it has been subsequently detected in rabbit populations throughout Asia, Europe, Australia, and the Americas, resulting in the death of millions of wild and domestic adult rabbits [3].

It is well known that the first step in viral infection is viral entry. However, a suitable cell culture capable of supporting authentic RHDV has not yet been established, thereby greatly impeding the progress of investigations into the mechanisms underlying the pathogenesis, translation, and replication of RHDV. Consequently, studies on the viral entry of RHDV have relied on the self-assembly of the capsid protein into virus-like particles when expressed in *Escherichia coli* or insect cells. RHDV has been shown to bind to oligosaccharides H type 2 and A type 2, which are histo-blood group antigens (HBGAs) expressed on the surfaces of cells lining the duodenal surface and trachea of rabbits, thereby presenting two possible viral entry points [5]. RHDV isolates from six different genetic groups bind specifically to different HBGAs, which act as attachment factors that facilitate infection [6,7].

Following attachment of RHDV to the cell surface, internalization by an unknown mechanism and desencapsidation occur, leading to the release of the viral genome into the cytoplasm of the host cell. In 2017, we successfully constructed and cultured a mutant RHDV (mRHDV) in RK-13 cells *in vitro*, which has a specific receptor-recognition motif (Arg-Gly-Asp) on the surface of the capsid protein that is characterized by two amino acid (aa) substitutions. mRHDV is recognized by the intrinsic membrane receptor (integrin α3β1) of RK-13 cells and then gains entry, replicates, and imparts apparent cytopathic effects [8].

In previous studies, we found that the RHDV capsid protein (VP60) was associated with many host proteins, including NCL, through affinity purification of infected cells [8]. NCL is a phosphoprotein that is ubiquitously and abundantly expressed in many growing eukaryotic cells and highly conserved during evolution, as it is involved in a remarkably large number of cellular activities [9]. Generally, NCL is mainly distributed in the nucleolus, but also exists in the nucleoplasm and cytoplasm, and on the cell surface, where its specific functions vary [10,11]. NCL controls a wide range of fundamental cellular processes, such as ribosome biogenesis, proliferation, and cell cycle regulation, and also plays important roles in the replication and intracellular trafficking of multiple viruses [12–16]. For example, the interaction between NCL and the 3′ untranslated region (UTR) of tombusvirus RNA has been shown to inhibit replication by interfering with the recruitment of viral RNA [17]. Similarly, the interaction between NCL and the UTRs of feline calicivirus [18] and poliovirus [19,20] stimulate translation of viral proteins. In addition, NCL binds to a protein of herpes simplex virus type 1 to facilitate exportation of US11 from the cell nucleus to the cytoplasm [21]. Notably, cell surface NCL is involved in viral infection by promoting viral attachment and internalization. For example, NCL acts as a receptor of human respiratory syncytial virus [22] and as a low-affinity receptor of human immunodeficiency virus [23]. NCL also mediates cellular attachment and internalization of enterovirus 71 [24]. Moreover, the internalization of multiple influenza A viruses, including H1N1, H3N2, H5N1, and H7N9, is reduced by suppression of NCL function and expression on the cell surface [25–29]. A recent study showed that NCL interacts with the capsid protein of dengue virus, suggesting a role in viral morphogenesis [30]. In addition, NCL is important for lyssavirus infection [31].

Here, we demonstrated that RHDV VP60 protein directly interacts with NCL. Of note, this interaction was found to play a key role in RHDV internalization. Furthermore, our data showed that NCL is involved in clathrin-dependent endocytosis, which is an important pathway of RHDV infection, by interacting with the C-terminal residues of clathrin light chain A (CLTA). Collectively, these findings further illustrate the molecular mechanism underlying RHDV infection.

## Results

### NCL is involved in RHDV internalization

**Fig 1:**
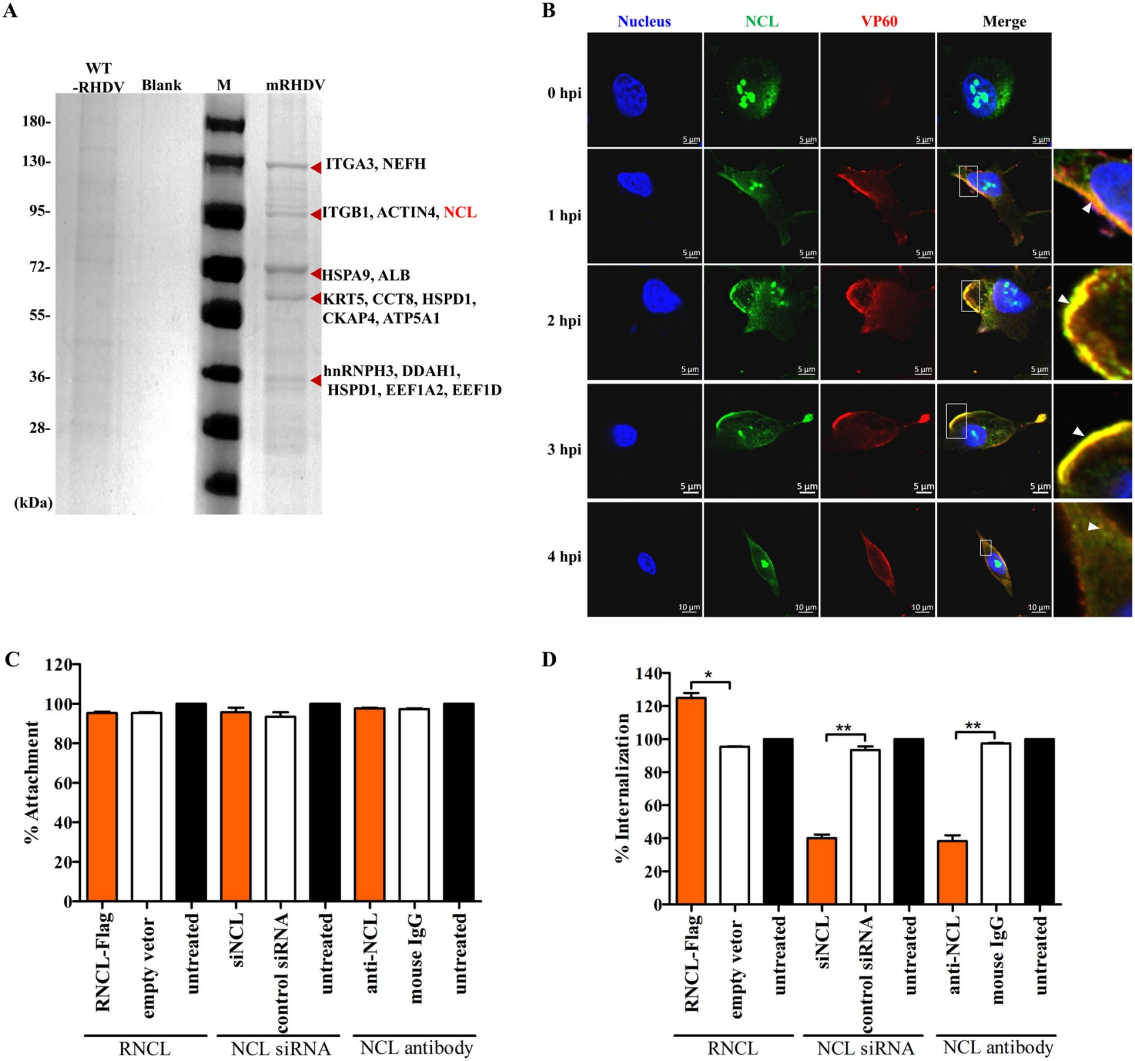
NCL is involved in RHDV internalization. **(A)** Screening of host proteins that interacted with the RHDV capsid protein. SDS-PAGE analysis of immunoprecipitation-purified host factors of RK-13 cells that interact with the mRHDV capsid protein. The red arrows indicate the bands identified by mass spectrometry. Lane M is the protein marker and the lane titled “blank” is the negative control (RK-13 cells not infected with mRHDV) [8]. **(B)** Immunofluorescence co-localization analysis of NCL (green) and VP60 (red). mRHDV-infected cells incubated with mAbs against NCL and VP60 at 0, 1, 2, 3, and 4 h, respectively. The small white boxes represent amplified random co-localization spots within the merged image, and the co-localization spots are indicated with white arrowheads. **(C)** The effect of changing the expression level of NCL on RHDV attachment. RK-13 cells were pre-transfected with Flag-tagged NCL plasmids or NCL siRNA for 24 h, or pre-incubated with anti-NCL mAb for 1 h at 37°C. The cells were then chilled and mRHDV was added at a MOI of 1. After viral attachment at 4°C for 2 h, the RK-13 cells were washed and lysed. The numbers of attached viral RNA copies were determined by qRT-PCR. Percent changes in VP60 RNA copy numbers were derived by comparing the number of VP60 RNA copies in the treated samples to those of the untreated RK-13 cells. The p3×FLAG-CMV-14 vector, non-specific siRNA, and mouse IgG were used as negative controls. **(D)** The effect of NCL on RHDV internalization. The treatment of cells in each group was the same as that for the attachment assay. RK-13 cells were incubated with mRHDV (MOI = 1) for 2 h at 4°C and then washed with chilled PBS. The temperature was then shifted to 37°C to facilitate viral internalization. At 30 min post infection, when RHDV internalization was proportionally increasing, the cells were washed in pre-chilled acidic PBS (pH = 2.5) to remove non-internalized viruses. After cell lysis, the amount of internalized mRHDV VP60 RNA copies was determined by qRT-PCR. The percentage of internalized VP60 copies was calculated as a ratio to the value obtained from untreated RK-13 cells. The student’s *t*-tests and analysis of variance were used for statistical analyses. *p < 0.05 and **p < 0.01. All experiments were conducted in triplicate, which produced similar results.

We previously reported a novel strategy for the construction of an mRHDV that could be recognized by host cells and grows well in RK-13 cells [8]. Furthermore, we performed affinity purification with RHDV capsid protein VP60 mAb from RK-13 cells, which were infected with mRHDV for 48 h, followed by mass spectrometry analysis. As shown in Fig 1A, many host proteins were associated with VP60, including NCL [8]. In this study, we found that RHDV and NCL were co-localized on the cell membrane surface at the early stage of mRHDV infection. This phenomenon was most apparent at 2–3 hours post-infection (hpi) (Fig 1B). Therefore, we hypothesized that NCL is important for RHDV entry.

To determine if NCL is required for RHDV infection, the expression level of NCL was changed by siRNA (S1 Fig) or transfection with plasmids (S2A Fig). For the attachment assay, RK-13 cells were pre-transfected with Flag-tagged NCL plasmids or NCL siRNA for 24 h, or pre-incubated with anti-NCL mAb for 1 h at 37°C. The cells were then chilled and mRHDV was added at a multiplicity of infection (MOI) of 1 (S3A Fig). After viral attachment at 4°C for 2 h, the RK-13 cells were washed and lysed. qRT-PCR determined the number of attached viral RNA copies. Percent changes in VP60 RNA copy numbers were derived by comparing the number of VP60 RNA copies in the treated samples to that of the untreated RK-13 cells. As shown in Fig 1C, mRHDV attachment to RK-13 cells was not obviously affected by changing or blocking NCL regulation.

Next, we investigated whether NCL was required for RHDV internalization. For the internalization assays, RK-13 cells were incubated with mRHDV (MOI = 1) for 2 h at 4°C and then washed with chilled PBS. The temperature was then shifted to 37°C to facilitate viral internalization. At 30 min post infection, when RHDV internalization was proportionally increasing (S3B Fig), the cells were washed in pre-chilled acidic PBS (pH = 2.5) to remove non-internalized viruses. After cell lysis, the amount of internalized mRHDV VP60 RNA copies was determined by qRT-PCR. As shown in Fig 1D, the percentage of mRHDV internalized in RK-13 cells was 40% or 38% after treatment with NCL siRNA or NCL Ab, respectively. In contrast, overexpression of NCL increased the percentage of internalized mRHDV by 25%.

Collectively, these data show that NCL is required during RHDV internalization, but not attachment.

### RHDV VP60 binds to the N-terminal domain of NCL

**Fig 2:**
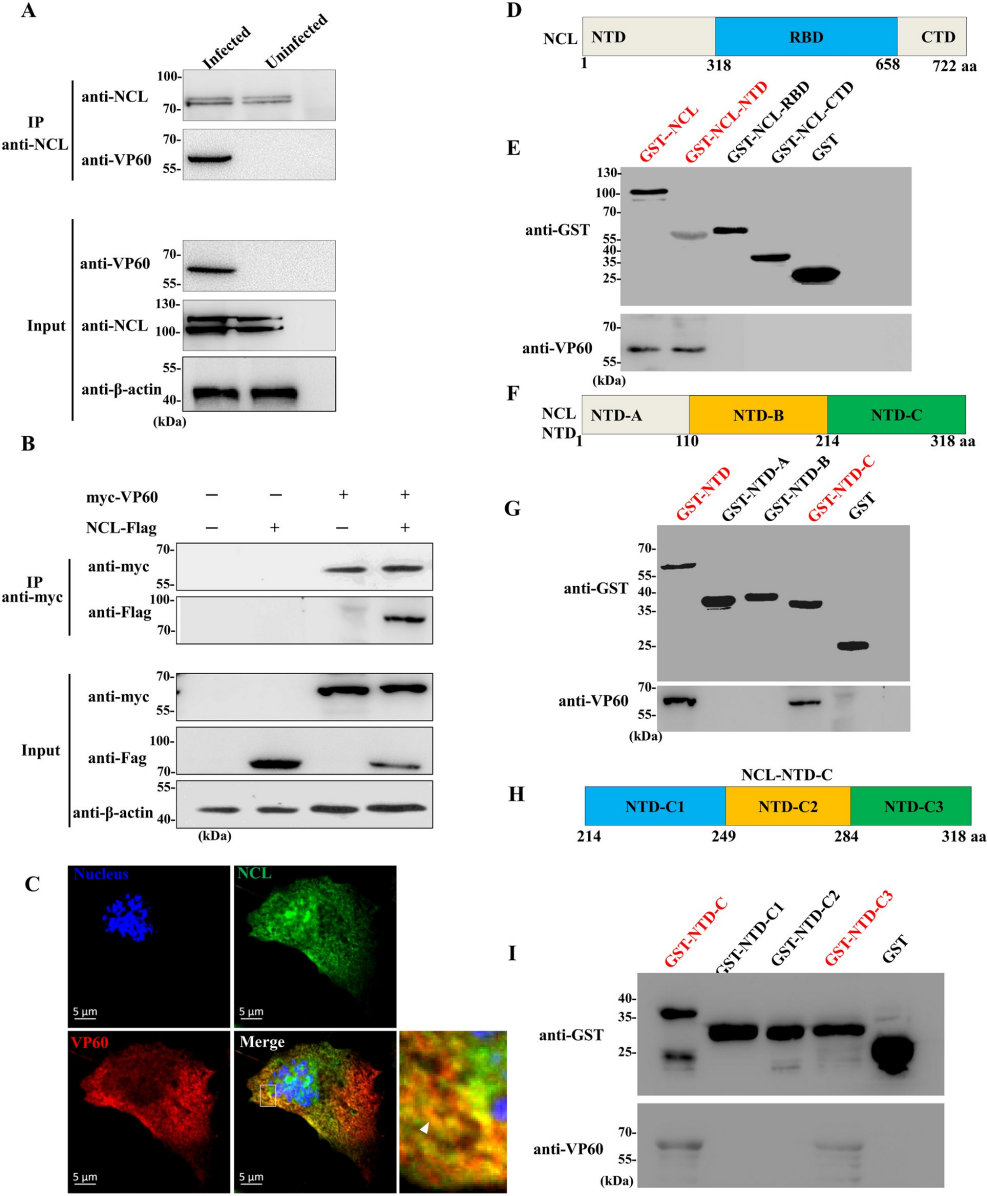
RHDV VP60 interacted with the N-terminal domain of NCL. **(A)** NCL binds to VP60 during RHDV internalization. An IP assay was performed on cell lysates using NCL mAb in RK-13 cells that were infected or uninfected with mRHDV, then immunoblotted with mAbs against NCL or VP60. β-actin was employed as an internal control. The cells uninfected with mRHDV served as negative control. **(B)** Validation of the interaction of VP60 with NCL by the Co-IP assay. RK-13 cells were co-transfected with plasmids expressing myc-VP60 and NCL-Flag. Cell lysates were prepared 48 h post-transfection and the proteins were immunoprecipitated using anti-myc mAb and then immunoblotted with mAbs against tag myc or Flag. β-actin was employed as an internal control. **(C)** Confocal microscopy analysis of NCL (green) and VP60 (red) in RK-13 cells co-transfected with myc-VP60 and NCL-Flag plasmids for 24 h with mAbs against NCL and VP60. The small white box represents amplified random co-localization spots within the merged image, and the co-localization spot is indicated with a white arrowhead. **(D)** Schematic representation of the strategy for constructing full-length GST-fusion proteins and the NTD, RBD, and CTD domains of NCL for the GST pull-down assay. The numbers indicate aa positions. **(E)** GST-fusion protein of NCL and its three domains were purified on glutathione-sepharose beads, and incubated in cell lysates obtained from RK13 cells transfected with the myc-VP60 plasimd for 48 h. After extensive washing, the binding of VP60 was determined by western blot analysis with an anti-VP60 mAb. The GST protein acted as a negative control. **(F)** Schematic illustration of the full-length and truncated forms of GST-NTD for the GST pull-down assay. The numbers indicate aa positions. **(G)** GST-NTD proteins were purified on glutathione-sepharose beads and incubated in RK-13 cells lysates prepared 48 h post-transfection with plasmids expressing myc-VP60. After extensive washing, bound VP60 was immunoblotted with anti-VP60 mAb. The GST protein acted as a negative control. **(H)** Schematic illustration of the full-length and truncated forms of GST-NTD-C for the GST pull-down assay. The numbers indicate aa positions. **(I)** Western blot analysis of glutathione affinity pull-down assays that were performed to map the locus of NCL binding. GST-fusions of various NCL-NTD fragments (NTD-C1, NTD-C2, and NTD-C3) were used as the bait and VP60 expressed in RK-13 cells as the prey. Binding of the VP60 protein was determined by western blot analysis with anti-VP60 mAb. The GST protein acted as a negative control.

Previous studies have reported that NCL is involved in the entry of dengue virus and enterovirus 71 by interacting with the capsid proteins [24,30]. The only capsid protein present in RHDV, VP60, is responsible for antigenicity and binding to host proteins, and plays a key role in RHDV entry [7]. To determine if NCL binds to VP60 during RHDV internalization, we assessed the interaction between NCL and VP60 in RK-13 cells in the presence and absence of mRHDV infection for 2h at 37°C. The results of an immunoprecipitation (IP) assay performed on cell lysates using NCL mAb showed that NCL and VP60 associated in infected cells (Fig 2A). Moreover, to determine whether NCL directly interacts with VP60, co-IP assays were employed with a myc mAb in RK-13 cells, which were co-transfected with pVP60-myc and pNCL-Flag eukaryotic expression plasmids. Western blot analysis using a mAb against the Flag tag showed a band corresponding to NCL in the myc co-IP assay, indicating a direct interaction between RHDV VP60 and NCL (Fig 2B). In addition, an IFA was performed using mAbs against VP60 and NCL in RK-13 cells co-transfected with pVP60-myc and pNCL-Flag plasmids for 24 h. As shown in Fig 2C, NCL was co-localized with RHDV VP60 in the RK-13 cell cytoplasm.

To characterize the critical domain of NCL for NCL-RHDV VP60 interactions, GST-fusion proteins corresponding to NCL and sub-fragments of NCL (GST-NCL, GST-NCL-NTD, GST-NCL-RBD and GST-NCL-CTD, respectively) were prepared for use as bait proteins in glutathione pull-down assays to determine their abilities to interact with the VP60 protein expressed in RK-13 cells (Fig 2D). The results of these assays showed that both GST-NCL and GST-NCL-NTD bound to RHDV VP60, but did not to GST-NCL-RBD, GST-NCL-CTD, and GST (Fig 2E). Subsequently, sub-fragments of NCL-NTD GST-fusion proteins (i.e., GST-NTD-A, GST-NTD-B, and GST-NTD-C) were prepared for use as bait proteins in the GST pull-down assay (Fig 2F). As shown in Fig 2G, both GST-NTD-C and GST-NCL-NTD bound to RHDV VP60, while the other proteins were undetectable. In addition, NCL-NTD-C was split into the fragments NTD-C1 (aa 214–249), NTD-C2 (aa 250–284), and NTD-C3 (aa 285–318), which were fused with GST and expressed (Fig 2H). The results of further pull-down assays showed that NTD-C3 and NTD-C bound to VP60, while the other proteins did not (Fig 2I).

Together these results suggest that RHDV VP60 directly and specifically interacts with the N-terminal residues 285–318 of NCL.

### A conserved sequence of RHDV VP60 is critical for binding of the NCL NTD domain

**Fig 3:**
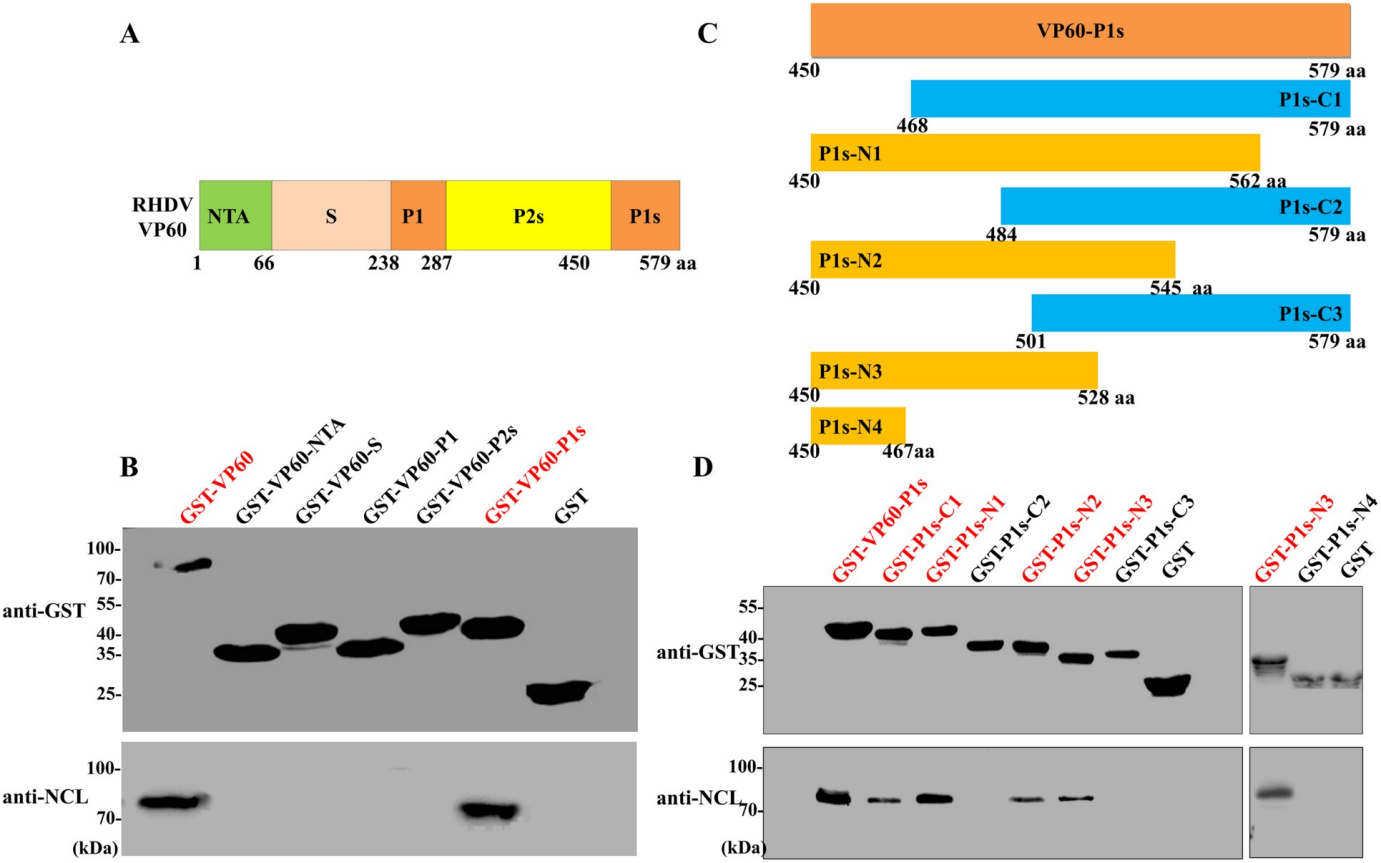
RHDV VP60 interacts with NCL residues 468–484. **(A)** Schematic diagram illustrating the construction of the full-length GST-fusion protein and the five domains of the VP60 protein for the GST pull-down assay. The numbers indicate the aa positions of each domain. **(B)** Western blotting analysis of glutathione affinity pull-down assays was performed to map the binding domain of the RHDV VP60 protein. GST-fusions of various VP60 domains (VP60-NTA, VP60-S, VP60-P1, VP60-P2s, and VP60-P1s) were used as the bait, while NCL expressed by RK-13 cells was used as the prey. Bound NCL was immunoblotted with anti-NCL mAb. The GST protein acted as a negative control. **(C)** Schematic illustration of the full-length and truncated forms of GST-VP60 P1s for the GST pull-down assay. The numbers indicate aa positions. **(D)** GST-P1s proteins were purified on glutathione-sepharose beads and incubated in RK-13 cell lysates containing the NCL protein. After extensive washing, NCL binding was determined by western blot analysis with anti-NCL mAb. The GST protein acted as a negative control.

RHDV VP60 is composed of three domains: N-terminal arm (NTA), shell (S), and protrusion (P). The P domain is further divided into the P1 and P2 sub-domains [32]. To identify functional areas of VP60 that interacts with NCL, the GST-VP60 fusion protein and its sub-fragments (i.e., GST-VP60-NTA, GST-VP60-S, GST-VP60-P1, GST-VP60-P2s, and GST-VP60-P1s) (Fig 3A) were prepared for use as bait proteins in GST pull-down assays to determine their abilities to interact with the NCL protein. As shown in Fig 3B, only GST-VP60-P1s and GST-VP60 bound to NCL. These results confirm that binding to NCL requires the P1s domain of VP60.

To further map the VP60 P1s segments responsible for the VP60-NCL interactions, GST-fusion proteins corresponding to sub-fragments of the VP60-P1s domain were prepared by removing 50 aa residues from the N- and C- terminals of VP60-P1s (i.e., GST-P1s-N1, GST-P1s-N2, GST-P1s-N3, GST-P1s-N4, GST-P1s-C1, GST-P1s-C2, and GST-P1s-C3) (Fig 3C). The results of a set of pull-down assays showed that GST-P1s-N1, GST-P1s-N2, GST-P1s-N3, GST-P1s-C1, and GST-P1s interacted with NCL, while the other proteins did not (Fig 3D). These findings indicate that RHDV VP60 interacts with NCL via aa residues 468–484.

**Fig 4:**
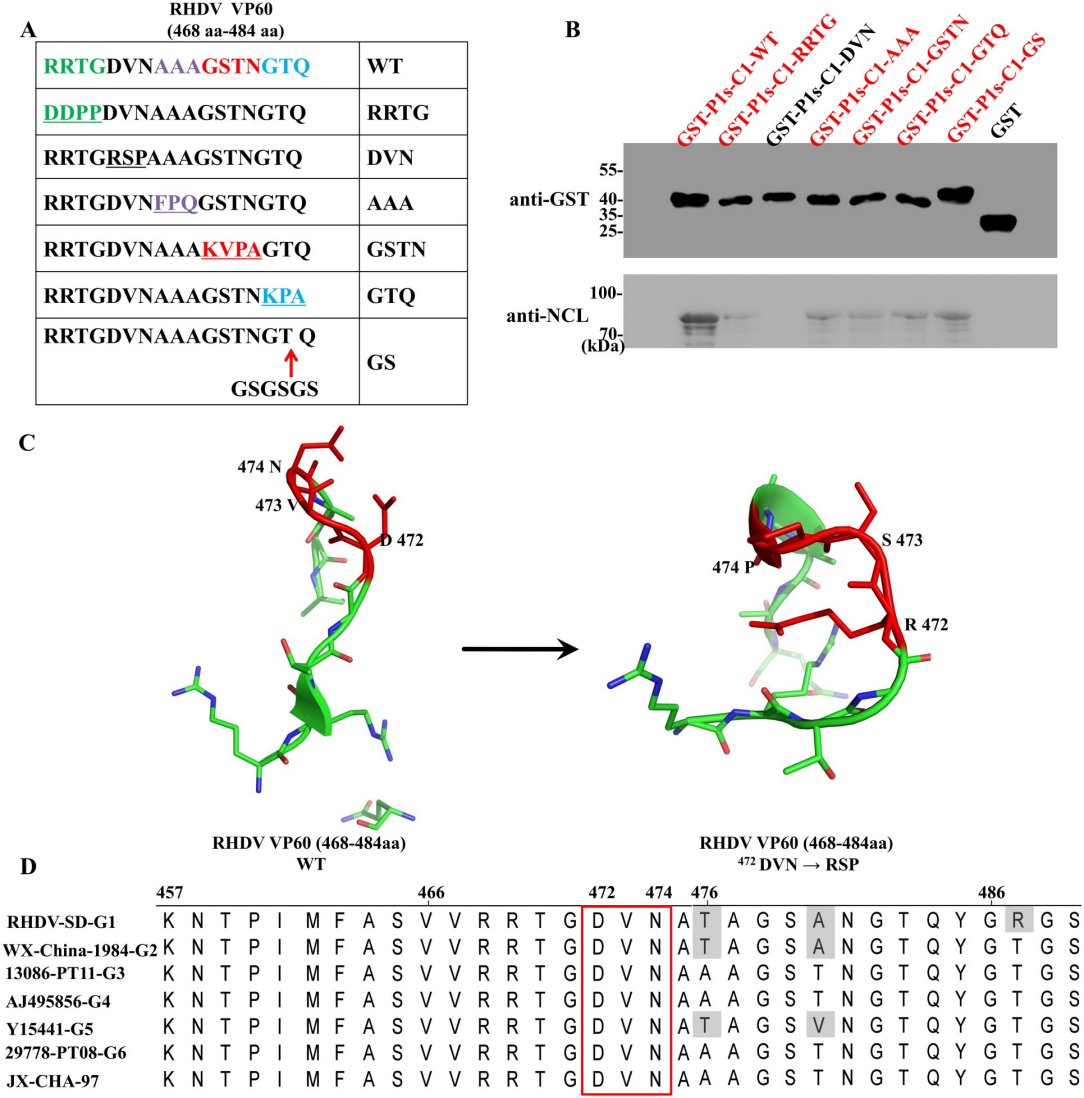
The P1s domain of the RHDV VP60 protein contains a conserved sequence motif that binds to the NCL NTD domain. **(A)** Sequence alignment showing the location of aa substitutions introduced into the GST-tagged VP60-P1s-C1. **(B)** Western blot analysis of the glutathione affinity pull-down assays using GST-tagged VP60-P1s-C1 mutants as the bait and NCL expressed in RK-13 cells as the prey. Bound NCL was immunoblotted with anti-VP60 mAb. The GST-P1s-C1 protein served as a positive control and the GST protein as a negative control. **(C)** Modeling of the RHDV VP60 P1s domain (aa 468–484). For clarity, the structure of the RHDV VP60 protein (aa 468–484) was obtained from the Protein Data Bank under the identification number 4egt.1.B (https://www.rcsb.org/). The structure of the ^472^DVN^472^RSP mutation, as predicted by the SWISS-MODEL online tool and based on homology molecules found in the Protein Data Bank. The DVN peptide is shown in red. **(D)** Alignments of representative aa sequences of the VP60 protein of the G1–G6 genogroups of RHDV. The representative strains used in the alignments were G1 RHDV-SD (GenBank accession no. Z29514.1), G2 RHDV strain WX/China/1984 (GenBank accession no. AF402614.1), G3 RHDV isolate 13086-PT11 (GenBank accession no. KJ683906.1), G4 RHDV isolate 00–13 (GenBank accession no. AJ495856), G5 RHDV isolate Hagenow (GenBank accession no. Y15441), G6 RHDV isolate 29778-PT08 (GenBank accession no. KJ683905), and RHDV strain JX/CHA/97 (GenBank accession no. DQ205345.1). Sequence alignment was performed with the ClustalW algorithm (http://www.clustal.org/). The conserved NCL-binding motif is boxed. Selected aa from this motif are indicated with RHDV VP60 numbering.

To pinpiont the key aa responsible for binding of the RHDV VP60 P1s domain with NCL, blocks of three or four aa substitutions were introduced within and beyond the conserved sequence motif. The following non-conservative substitutions to the GST-tagged RHDV VP60 protein were made: ^468^RRTG^468^DDPP, ^472^DVN^472^RSP, ^475^AAA^475^FPQ, and ^482^GTQ^482^KPA. Furthermore, the sequence GSGSGS was inserted after aa residue 483 (Fig 4A). It has been reported that GSGSGS is a flexible peptide for use in different expression systems to separate functional proteins [33]. The wild-type and mutant RHDV VP60 P1s proteins were used as bait proteins in the GST pull-down assays to determine their abilities to bind to NCL. As shown in Fig 4B, the ^472^DVN^472^RSP mutation was not able to bind to NCL, while the binding capacities of the other mutations to the residues at positions 468–484 were reduced to different extents. Moreover, as predicted with the SWISS-MODEL online tool (https://swissmodel.expasy.org/), wild-type DVN peptides formed a structure similar to a “claw,” which provides a structural basis for interactions with NCL. However, once the key aa were changed (DVN mutated to RSP), the “claw” structure is broken and loses the ability to bind to NCL (Fig 4C). These results indicate that this “claw” structure is the structural basis for the interaction between VP60 and NCL. In addition, analysis of the VP60 sequence of the G1–G6 genotypes of RHDV showed that the DVN motif was highly conserved (Fig 4D).

These observations confirm that RHDV VP60 interacts with NCL via the DVN (Asp-Val-Asn) motif, which is a conserved sequence in RHDV.

### The interaction between VP60 and NCL plays a key role in RHDV internalization

**Fig 5:**
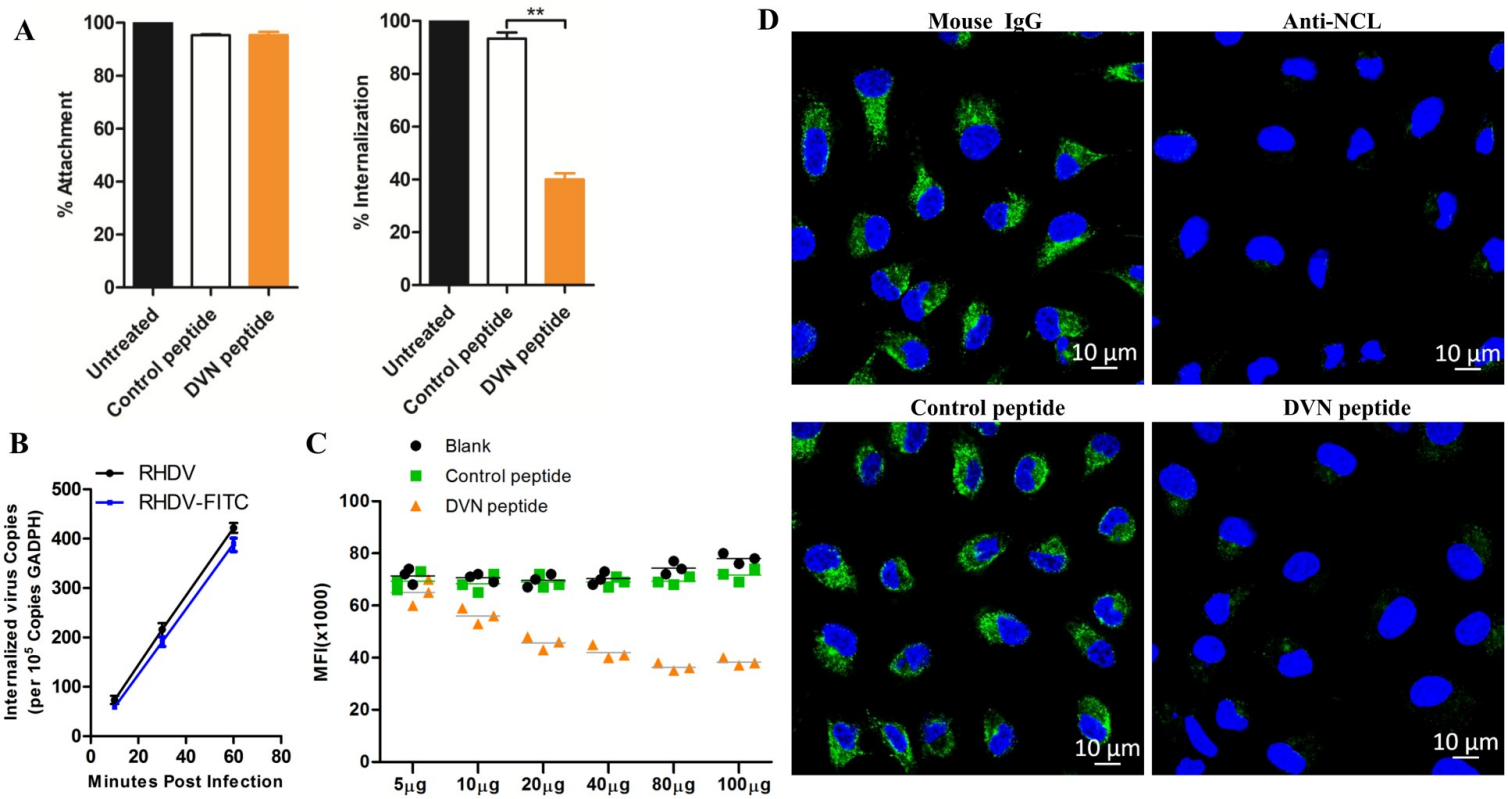
Synthetic DVN peptides significantly inhibit RHDV internalization. **(A)** RK-13 cells were pretreated with DVN or control peptides at a concentration of 100 μg/mL for 6 h. Then, the attachment and internalization assays were performed with mRHDV-infected cells (MOI = 1) and the cells harvested for RNA analysis. The percentage of RHDV VP60 copies was calculated as a ratio to the value obtained from untreated RK-13 cells. The control peptide was employed as a negative control. **(B)** Kinetics of mRHDV internalization into RK-13 cells. Internalization of mRHDV (MOI = 1) or mRHDV-FITC into RK-13 cells was assessed by mRHDV incubation for 10, 30, and 60 min at 37°C. The amount of internalized virus was determined by qRT-PCR and expressed as VP60 RNA copies per 100,000 of GADPH. **(C)** RK-13 cells were pretreated with the DVN and control peptides at concentrations of 5, 10, 20, 40, 80, and 100 μg/mL for 6 h, respectively. mRHDV-FITC-infected cells (MOI = 1) were then used for internalization assays and harvested for flow cytometry analyses. The control peptide was employed as a negative control. The data are representative of the means and standard deviations (error bars) of three samples per group (**p < 0.01). All experiments were repeated at least three times with consistent results. **(D)** RK-13 cells were pretreated with the DVN peptides (100 μg) or anti-NCL mAb (50 μg) for 6 h. Then, the cells were used for the internalization assay with mRHDV-FITC (MOI = 1) and subsequent immunofluorescence analysis. The RHDV virion is green and cell nucleus in blue. Mouse IgG and control peptides were employed as negative controls.

The above results indicate that NCL was required during RHDV internalization and interacted with the RHDV capsid protein (VP60). Therefore, we speculated that NCL is involved in RHDV internalization via binding to VP60. To verify this hypothesis, we blocked the binding site of NCL-VP60 with a synthesized DVN peptide (RRTGDVNAAAGSTNGTQ) and examined its effect on RHDV entry. The results of the attachment and internalization assays showed that mRHDV internalization was drastically reduced in RK-13 cells treated with the DVN peptide, with only 40% internalization, but there was no obvious effect on mRHDV attachment (Fig 5A).

To image the internalization of single RHDV particles in live cells, the fluorescent dye Alexa Fluor 488 was conjugated to purified virions. This labeling process did not significantly reduce the viral titer, as measured by the qRT-PCR assay (Fig 5B). Alexa Fluor 488-conjugated mRHDV (RHDV-FITC) was used to evaluate the efficiency of viral internalization in response to different doses of the DVN peptide as well as a control peptide (RHDV VP60 residues 434–450: VTYTPQPDRIVTTPGTP). RK-13 cells were pretreated with DVN peptide or a control peptide at a concentration of 5, 10, 20, 40, 80, or 100 μg/mL, respectively for 6 h, and then incubated with RHDV-FITC (MOI = 1) for 2 h at 4°C and washed with chilled PBS. The temperature was then shifted to 37°C to facilitate viral internalization. At 30 min post infection, when RHDV internalization was proportionally increasing, the cells were washed in pre-chilled acidic PBS (pH = 2.5) to remove non-internalized viruses. The amount of internalized mRHDV was quantitated by flow cytometry. As shown in Fig 5C, with the increase in the DVN peptide, the amount of RHDV internalized in RK-13 cells was significantly reduced, and when the cells were treated with DVN peptide (80 μg/mL), the amount of virus in the treated cells was only about half of the control group. In addition, fluorescence microscopy also showed that the amount of internalized mRHDV was drastically reduced in RK-13 cells treated with either the DVN peptide or NCL mAb (Fig 5D).

These results suggest that blocking the binding site of the interaction between VP60 and NCL resulted in a significant reduction in RHDV internalization.

### Clathrin-dependent endocytosis is involved in RHDV internalization

**Fig 6:**
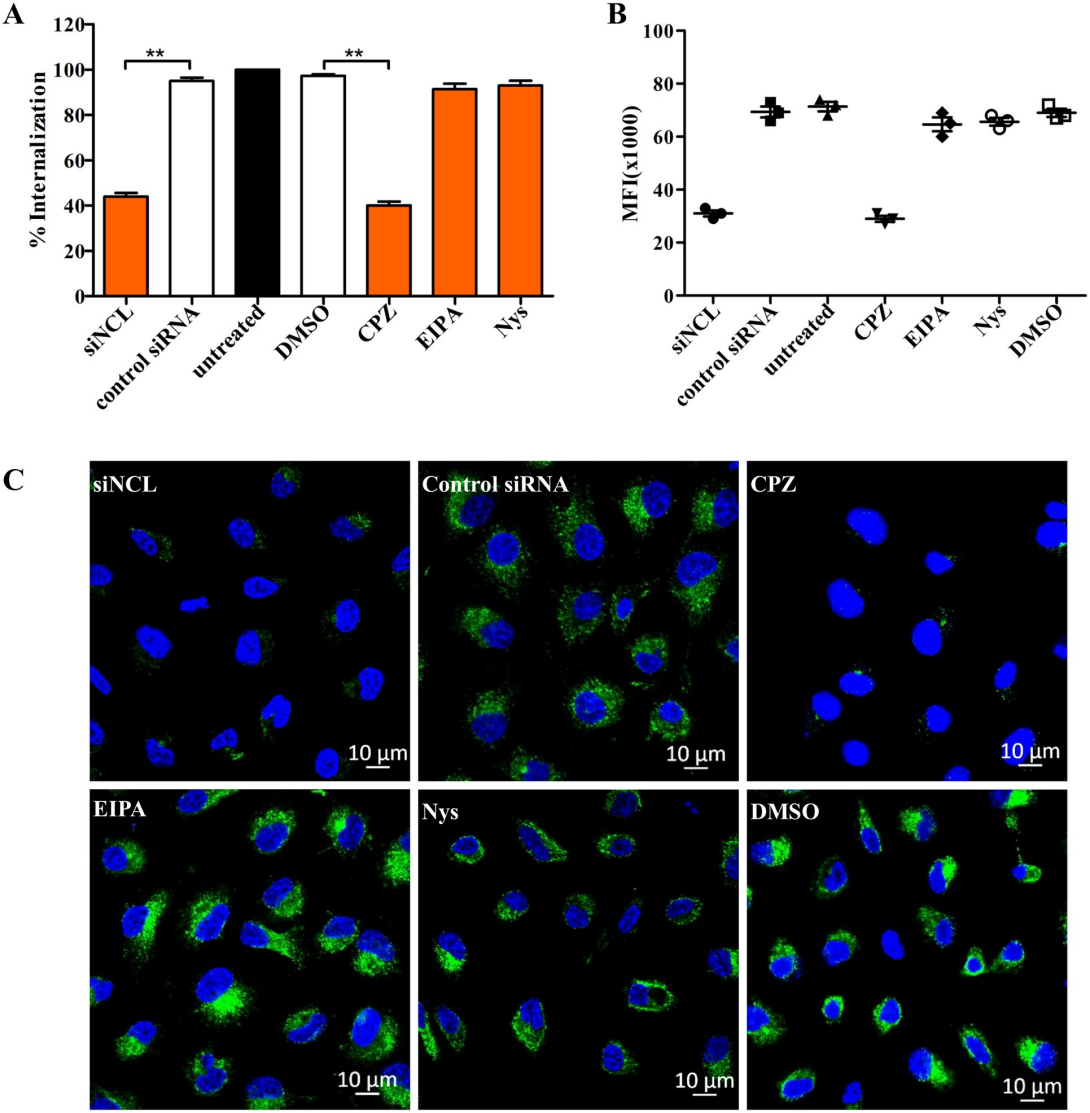
Clathrin-dependent endocytosis is involved in RHDV internalization and is regulated by NCL. **(A)** Clathrin-dependent endocytosis is involved in RHDV internalization. RK-13 cells of three groups were pretreated with CPZ (30 mM), EIPA (50 mM), or Nys (100 mM) for 2 h. Another group of cells was transfected with NCL siRNA (100 nM) for 24 h. Cells were then prompted to internalize mRHDV (MOI = 1) and harvested for RNA analysis. The percentage of RHDV VP60 copies was calculated as a ratio to the value obtained from untreated RK-13 cells. DMSO and non-specific siRNA were used as negative controls. The data are presented as the mean and standard deviation (error bars) of three samples per group (**p < 0.01). All experiments were repeated at least three times and showed consistent results. **(B)** mRHDV-FITC (MOI = 1) infected RK-13 cells, treated with procedures described above, were subjected to the internalization assay and harvested for flow cytometry analysis. **(C)** RK-13 cells of three groups were pretreated with CPZ (30 mM), EIPA (50 mM), or Nys (100 mM) for 2 h. Another group cells was transfected with NCL siRNA (100 nM) for 24 h. Then, the cells were used for the internalization assay with mRHDV-FITC (MOI = 1) and subsequent immunofluorescence analysis. The RHDV virion is green, cell nucleus is blue. DMSO and control siRNA were employed as negative controls.

It is well known that viruses typically make use of various pinocytic mechanisms of endocytosis that serve the cell by promoting the uptake of fluid solutes, and small particles. The mechanisms include clathrin-dependent endocytosis, the most commonly used mechanism for virus, macropinocytosis, a transient, ligand-induced, actin-dependent mechanism, and lipid raft-dependent endocytosis, a lipid raft-dependent mechanism mainly used by polymaviruses [34]. A previous study reported that RHDV VLPs use clathrin-dependent endocytosis to enter mouse and human dendritic cells [35]. However, the mechanism by which RHDV enters rabbit cells remains unclear. In order to determine the type of endocytosis involved in RHDV internalization, a series of experiments were conducted to examine RHDV entry into RK-13 cells. First, the cells were treated with 30 mM CPZ, an inhibitor of clathrin-dependent endocytosis, which inhibits clathrin triskelions assembly, 100 mM Nys, an inhibitor of lipid raft-dependent endocytosis, which disrupts membrane lipid rafts, 50 mM 5-EIPA, an inhibitor of macropinocytosis, which is a selective blocker of Na^+^/H^+^ pump exchanger, or DMSO, respectively. The effect of inhibitors on RHDV-FITC internalization was quantified by qRT-PCR, flow cytometry, and fluorescence microscopy. The results showed that CPZ, as well as NCL siRNA, inhibited RHDV internalization, as the percentage of internalization was about 40%, but not Nys or EIPA (Fig 6A–6C), suggesting that clathrin-dependent endocytosis is involved in the process of RHDV entry into rabbit cells. Our results are consistent with those of previous studies, suggesting that clathrin-dependent endocytosis is involved in RHDV internalization.

### NCL plays a key role in clathrin-dependent endocytosis by interacting with the C-terminal domain of CLTA

Previous studies have shown that NCL is required for clathrin-dependent endocytosis in human and murine cells [27,36]. Here, FITC-conjugated ligands were used to evaluate the efficiency of cargo uptake by siRNA-treated and untreated RK-13 cells. The effect of NCL siRNA on all endocytic pathways was quantified by flow cytometry. The results showed that NCL siRNA inhibited the uptake of transferrin and EGF, which were used as representative markers of clathrin-dependent endocytosis [37], but had no effect on the uptake of the representative markers of the lipid raft-dependent endocytosis (CD4 [38]) and macropinocytosis (dextran [39]) (S4A–S4D Fig). These results showed that NCL also plays a key role in clathrin-dependent endocytosis in RK-13 cells.

**Fig 7:**
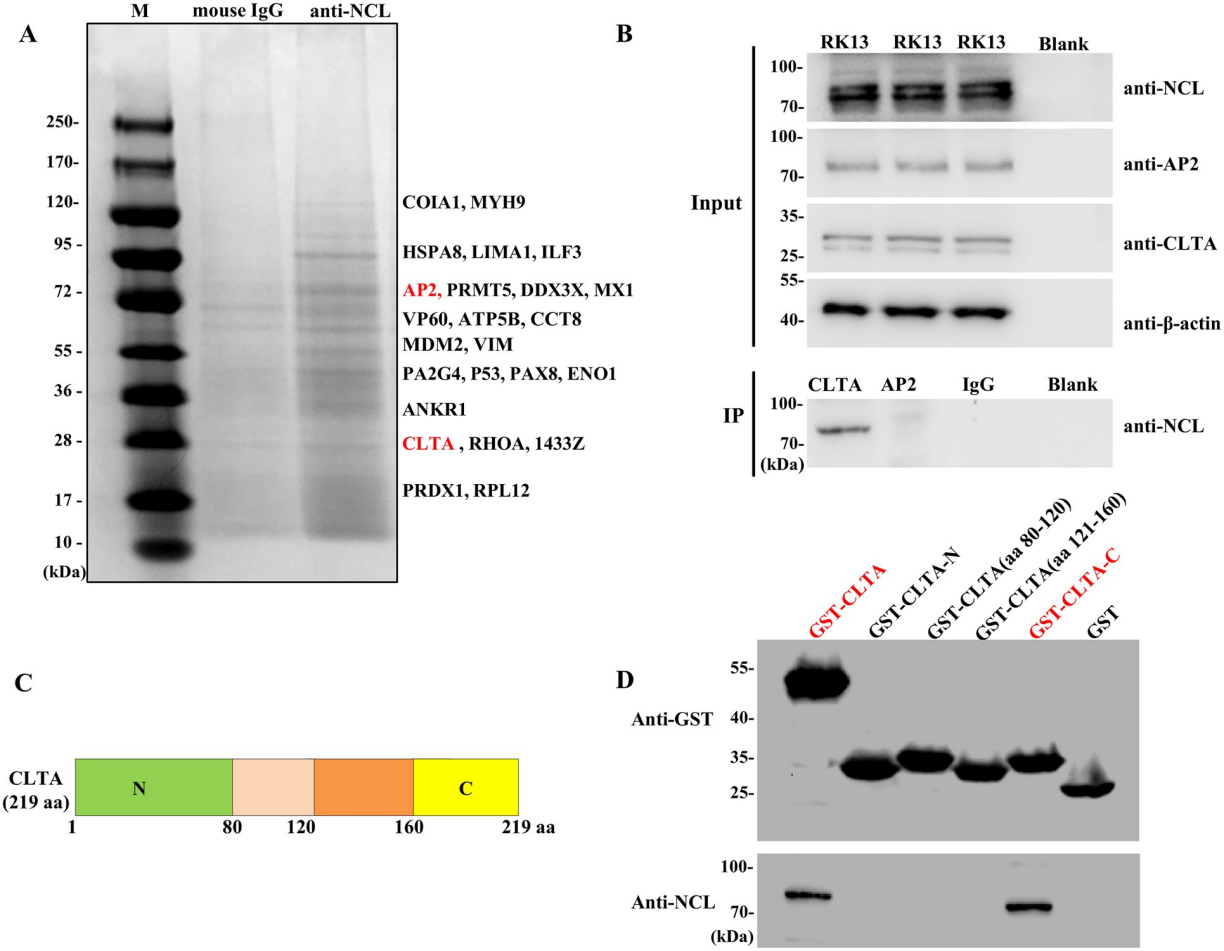
NCL binds to the C-terminal domain of CLTA. **(A)** Screening of host proteins that interacted with the NCL protein. SDS-PAGE analysis of immunoprecipitation-purified host factors of RK-13 cells that interact with the NCL protein was performed with NCL mAb. The bands were identified by mass spectrometry. In addition, M is a protein marker and the negative control was cell lysates immunoprecipitation-purified with mouse IgG. **(B)** Validation of the interaction between CLTA and NCL with the IP assay. Proteins in the lysates of RK-13 cells were immunoprecipitated with anti-CLTA mAb, anti-AP2 mAb, or mouse IgG, and then immunoblotted with anti-NCL mAb. β-actin was employed as an internal control. **(C)** Schematic illustration of the full-length and truncated forms of GST-CLTA for the GST pull-down assay. The numbers indicate aa positions. **(D)** GST-CLTA proteins were purified on glutathione-sepharose beads and incubated in RK-13 cells lysates containing the NCL protein. After extensive washing, the binding of NCL was determined by western blot analysis with anti-NCL mAb. The GST protein acted as a negative control.

However, the molecular mechanism underlying NCL involvement in clathrin-dependent endocytosis remains unclear. To elucidate the molecular mechanism, RK-13 cell lysates were used for affinity purification with NCL mAb. Briefly, the eluted protein complexes were resolved by SDS-PAGE and the protein bands were visualized by silver staining and identified by mass spectrometry analysis. As shown in Fig 7A, many host proteins were bound to NCL, including adaptor protein 2 (AP2) and CLTA, which are necessary for clathrin-dependent endocytosis. CLTA participates in several membrane traffic pathways involving both clathrin and actin by binding with actin-organizing huntingtin-interacting proteins [40]. Moreover, IP experiments were performed with an NCL mAb in RK-13 cells. Western blot analysis using a mAb against AP2 or CLTA showed a band corresponding to CLTA, but not AP2, indicating an interaction between CLTA and NCL (Fig 7B). Furthermore, the crucial domain of the interactions between NCL and CLTA was investigated using the glutathione pull-down assays. The GST-fusion proteins corresponding to CLTA and sub-fragments of CLTA (GST-CLTA, GST-CLTA-N, GST-CLTA (aa 81–120), GST-CLTA (aa 121–160), and GST-CLTA-C, respectively) were prepared for use as bait proteins to determine their abilities to interact with the NCL protein expressed in RK-13 cells (Fig 7C). These results showed that CLTA-GST and GST-CLTA-C bound to NCL, but no GST-CLTA-N, GST-CLTA(aa 81–120), GST-CLTA(aa 121–160) or GST (Fig 7D).

Taken together, these results suggest that NCL plays a key role in clathrin-dependent endocytosis by directly and specifically interacting with CLTA via the C-terminal aa residues 161–219.

### The interaction between CLTA and NCL plays an important role in RHDV internalization

**Fig 8:**
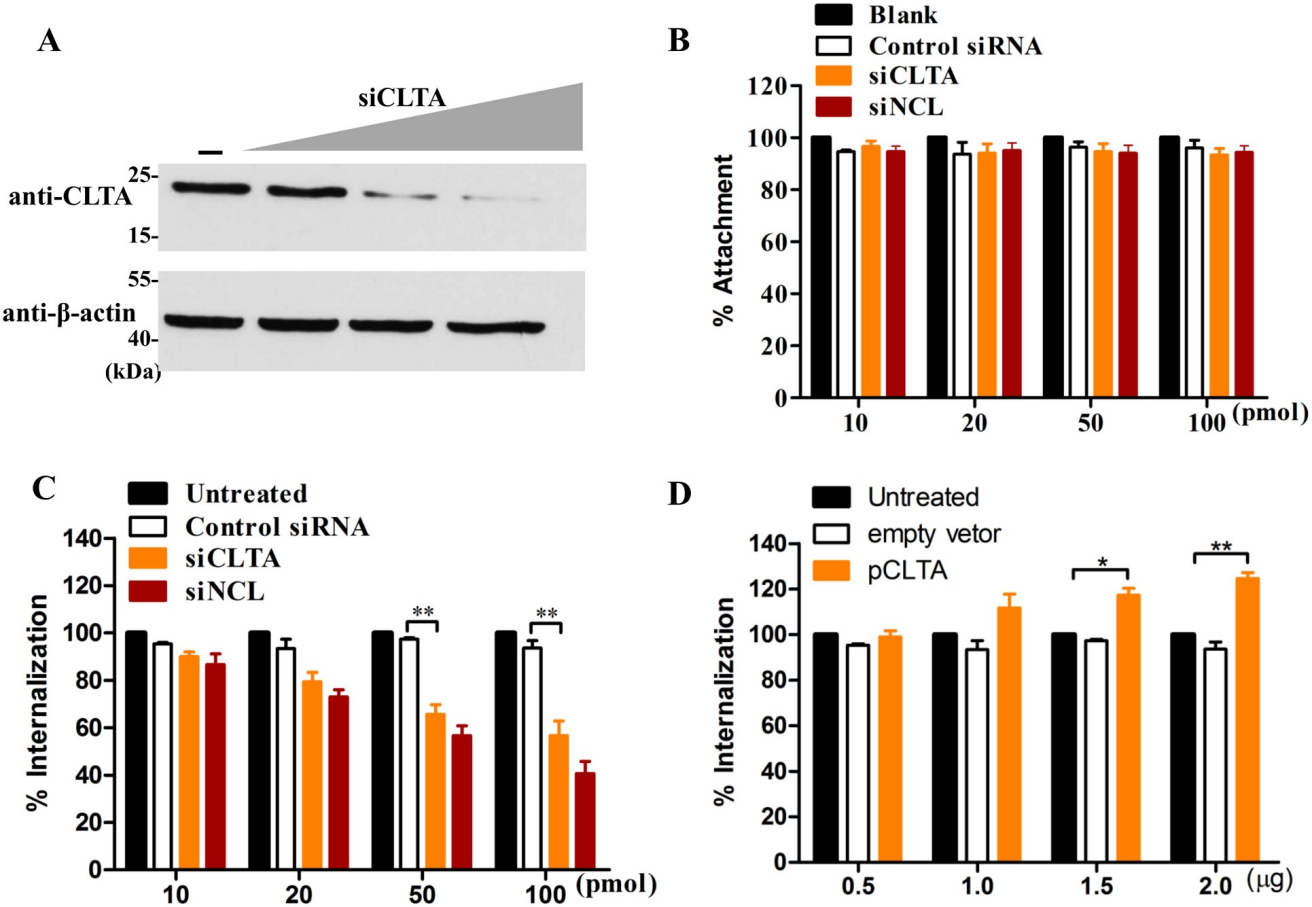
The interaction between CLTA and NCL plays an important role in RHDV internalization. **(A)** The expression level of CLTA was determined by western blot analysis with anti-CLTA mAb. Cell lysates were obtained from RK-13 cells transfected with CLTA siRNA (10, 20, 50, or 100 nM) for 24 h. β-actin was employed as an internal control. **(B)** The effect of CLTA siRNA on RHDV attachment. Before the addition of RHDV (MOI = 1) for 2 h at 4°C, RK-13 cells were transfected with CLTA siRNA. Non-specific siRNA was used as a control. siNCL acted as a positive control. The percentage of attached RHDV VP60 copies was calculated as a ratio to the value obtained from untreated RK-13 cells. **(C-D)** The effect of CLTA on RHDV internalization. Before the addition of RHDV (MOI = 1) for 2 h at 4°C, RK-13 cells were transfected with CLTA siRNA (C) or the pCLTA plasmid (D). Non-specific siRNA or pCMV-Myc was used as negative control. siNCL acted as a positive control. The percentage of internalized VP60 copies was calculated as a ratio to the value obtained from untreated RK-13 cells. The student’s *t*-tests and analyses of variance were used for statistical analyses, *p < 0.05 and **p < 0.01. The experiments were conducted in triplicate and produced similar results.

The results of the above studies showed that NCL is involved in RHDV internalization by binding to VP60. In addition, clathrin-dependent endocytosis is involved in RHDV internalization and NCL participates in clathrin-dependent endocytosis by interacting with CLTA. To investigate the role of the interaction between CLTA and NCL in RHDV internalization, the expression level of CLTA was changed by siRNA (Fig 8A) or myc-CLTA plasmid (S2B Fig) transfection. The results of the attachment and internalization assays showed that mRHDV internalization was reduced in RK-13 cells treated with CLTA or NCL siRNA (Fig 8C), and increased by overexpression of CLTA (Fig 8D), athough there was no obvious influence on mRHDV attachment (Fig 8B). Of note, the effect on mRHDV internalization is dependent on the dose of CLTA siRNA, NCL siRNA or the myc-CLTA plasmid. The percentage of mRHDV internalization in RK-13 cells was about 41%, 56% and 125%, following treatment with CLTA siRNA (100 pmol), NCL siRNA (100 pmol) or myc-CLTA (2 μg), respectively (Fig 8C and 8D). These data showed that CLTA is involved in RHDV internalization, confirming that the interaction between CLTA and NCL plays an important role in RHDV internalization.

### The DVN peptide provides some protection against RHDV infection in rabbits

**Fig 9:**
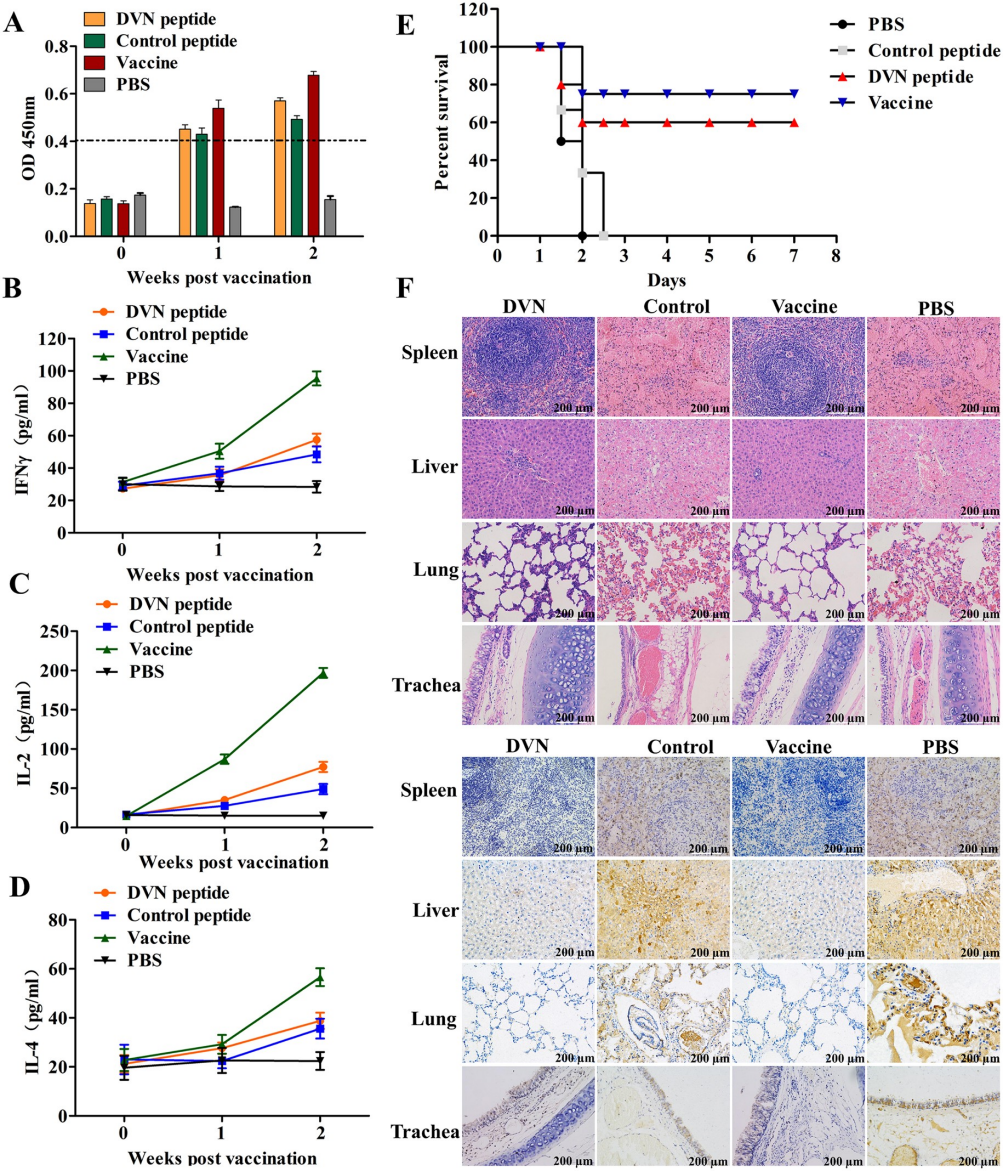
DVN peptides provide some protection to rabbits against RHDV infection. **(A)** RHDV-specific Ab responses of rabbits to the DVN peptide. Twenty 8-week-old rabbits that were seronegative for RHDV were randomly distributed into four groups (n = 5/group) and immunized with the DVN peptide (1 mg), control peptide (1 mg), commercial inactivated RHDV vaccine, or PBS, respectively. Rabbit sera from different groups were collected at 0, 1, and 2 weeks post-immunization, and subjected to an indirect ELISA. Optical density (OD) values at a wavelength of 450 nm were measured in quadruplicate. **(B)** IFN-γ production by lymphocytes in immunized rabbits. **(C)** IL-2 production by lymphocytes in immunized rabbits. **(D)** IL-4 production by lymphocytes in immunized rabbits. Serum levels of IL-2, IL-4, and IFN-γ were measured by an ELISA. Samples were tested in quadruplicate and data are presented as the mean ± standard deviation. **(E)** The survival of rabbits infected with RHDV JX/CHA/97. All rabbits were challenged intramuscularly with 100 LD_50_ of RHDV at 21 days post-immunization and subsequently clinically examined daily for 7 days. **(F)** The results of postmortem histopathological and immunohistochemical analyses. All animals were necropsied and organs were collected. For the histopathological analysis, samples were fixed in 10% neutral buffered formalin solution, sectioned, and stained with hematoxylin and eosin. Alternatively, immunohistochemical analysis was conducted with anti-VP60 mAb.

The above experimental results were obtained with RK-13 cells infected with mRHDV. In order to assess the role of NCL-VP60 interaction during infection with wild-type RHDV, rabbits were immunized with the DVN peptide, control peptide, commercial inactivated RHDV vaccine, or PBS. The VP60-specific Ab responses of immunized rabbits were determined using an indirect ELISA. As shown by the results presented in Fig 9A, the Ab titers of VP60 from the DVN peptide, control peptide, and commercial vaccine groups gradually increased. Statistically, the Ab titers were significantly higher (*p* < 0.05) in the DVN peptide, control peptide, and commercial vaccine groups than in the PBS group at 0, 1 and 2 weeks post-immunization. Simultaneously, in order to investigate cell-mediated immune responses, the amounts of IFN-γ, IL-2, and IL-4 were measured by ELISA. As shown in Fig 9B–9D, there was no significant difference in the levels of IFN-γ, IL-2, and IL-4 over time between the DVN peptide and control peptide groups. However, the levels of IFN-γ, IL-2, and IL-4 were significantly higher (*p* < 0.05) in the commercial vaccine group than in the PBS group at 1 and 2 weeks post-immunization. These results indicated that the DVN peptide was unable to activate the cellular immune response in rabbits. At 21 days post immunization, all rabbits were challenged intramuscularly with wild-type RHDV. As shown in Fig 9E, the survival rates of rabbits against virulent RHDV in the DVN peptide and commercial vaccine groups were 60% and 80%, respectively. However, all rabbits in the control peptide group and PBS group (negative control) died within 48–72 hpi with virulent RHDV. These negative control animals exhibited clinical symptoms of RHDV infection. Moreover, histopathological analysis indicated that typical pathological changes, such as diffuse bleeding of tissues and organs, were observed in the control peptide and PBS groups, but the tissues and organs of rabbits in the DVN peptide and commercial vaccine groups showed no pathological changes. Similarly, immunohistochemical analysis indicated large amounts of RHDV virions in the tissues of rabbits in the control and PBS groups, but not in the DVN peptide and commercial vaccine groups (Fig 9F).

These data show that the DVN peptide provides partial protection against RHDV in rabbits, suggesting that the interaction between VP60 and NCL plays a key role in RHDV internalization.

## Discussion

NCL is well known as a multifunctional protein that is mainly localized in the nucleolus, but is also found in the nucleoplasm and cytoplasm, as well as on the cell membrane [10]. The multi-functionality of NCL mainly results from its multi-domain structure. Sequence comparison of different species revealed a high degree of evolutionary conservation of this large protein, and biophysical and biochemical studies have shown that NCL is composed of three main structural domains: the N-terminal domain, the central domain, and the C-terminal domain [41]. The N-terminal domain of NCL contains acidic regions, rich in glutamic acid and aspartic acid, which are the sites of phosphorylation, and participate in the transcription of rRNA and interact with components of the pre-rRNA processing complex [16]. The central domain of NCL contains four RNA-binding domains (RBDs), also known as RNA-recognition motifs, which are involved in a variety of biological processes, including RNA packaging, pre-mRNA splicing, poly-A tail synthesis and maturation, translational control, and mRNA stability [11]. The C-terminal region of NCL contains a glycine- and arginine-rich domain, through which NCL interacts with target mRNAs and proteins, including ribosomal proteins [9].

NCL is involved in several cellular functions, including ribosome biogenesis, DNA and RNA metabolism, cell proliferation, apoptosis regulation, and stress responses [42]. Moreover, increasing evidence suggests that NCL is also involved in the pathogenesis of many viruses. For viral infection, cell surface NCL has been shown to be involved by promoting either initial attachment of the virus to the cell surface or entry into host cells. For instance, NCL was found to serve as a cellular receptor during infection with human respiratory syncytial virus [22]. Cell surface NCL also mediates the binding and internalization of enterovirus 71 [43]. By targeting NCL on the cell surface, HB-19 pseudo-peptides inhibit attachment of human immunodeficiency virus to the cell surface as well as subsequent viral entry into the host cell [44]. Furthermore, cell surface NCL ligands, such as midkine and lactoferrin, have similar effects during viral infection [45]. In addition, NCL serves as a conserved cellular factor that is required for cell entry of multiple influenza A viruses, including H1N1, H3N2, H5N1, and H7N9. Suppression of the expression or function of cell surface NCL by siRNA or blocking Abs substantially reduced internalization of the influenza virus [9,25,26,29].

In this study, we found that NCL is also involved in RHDV infection and plays a key role in RHDV internalization. It has been reported that HBGAs are functional receptors of RHDV and play key roles in viral attachment to the cell surface [5]. Data derived from the three-dimensional structure of RHDV suggest that HBGAs mainly bind to the P region of the RHDV capsid protein, thereby triggering RHDV infection of host cells [7,9]. However, the molecular mechanism underlying RHDV invasion of host cells remains unknown. The results of the present study showed that NCL was involved in RHDV internalization. In other words, following RHDV binding to the cell receptor (HBGA), the viral internalization process is completed with the help of NCL, which acts as a bridge that connects RHDV VP60 and CLTA. In this study, the NTD domain of NCL was found to bind to the RHDV VP60 protein via a conserved sequence at the P1s domain of VP60, and the internalization efficiency of RHDV was significantly inhibited by blocking these interactions. This finding was also confirmed by the animal experiments. Rabbits were partially protected from RHDV assault (~ 60%) after inoculation with synthetic DVN peptides. In the designed experiment, activation of the humoral immune response of rabbits and resulted in the production of specific Abs against DVN. However the cellular immune response was very low. We speculated that the specific Abs induced by DVN peptides blocked the internalization of RHDV, and provided protection to the rabbits.

However, the role of NCL in RHDV infection differs from that of other viruses. The results of this study showed that NCL does not directly mediate viral entry into cells as a functional receptor for RHDV, but still plays a key role in the RHDV internalization stage, as NCL was found to mediate the entry of RHDV into the cells by clathrin-dependent endocytosis via binding to the C-terminus of CLTA. It is well known that clathrin-dependent endocytosis is a classical endocytic pathway that transports extracellular substances into the cell, and most enveloped and non-enveloped viruses enter the host cell by this endocytic pathway [34]. Clathrin consists of light and heavy chains, and forms a triangular complex structure. When an extracellular substance binds to the receptor, the clathrin complex is recruited to the cell membrane surface in response to endocytic signaling and pulls the substance down to the cell membrane, where it becomes trapped and forms clathrin-invaded pits. Under the action of dynein, the invaginated pits leave the cell membrane and form clathrin-coated vesicles that eventually pass the endocytosed material to endosomes [40]. Previous studies have shown that NCL is involved in the internalization of the EGF receptor, which is mediated by clathrin-dependent endocytosis [46]. Also, NCL is involved in clathrin-dependent endocytosis through interactions with the C-terminus of CLTA. This discovery should prove helpful to further understand clathrin-dependent endocytosis. Inhibition of clathrin-dependent endocytosis by drugs inhibited the internalization of RHDV, which further demonstrated that clathrin-dependent endocytosis indeed plays an important role in RHDV infection. Together, these results demonstrated that RHDV is internalized by the host cell via clathrin-dependent endocytosis, which is mediated by NCL.

**Fig 10:**
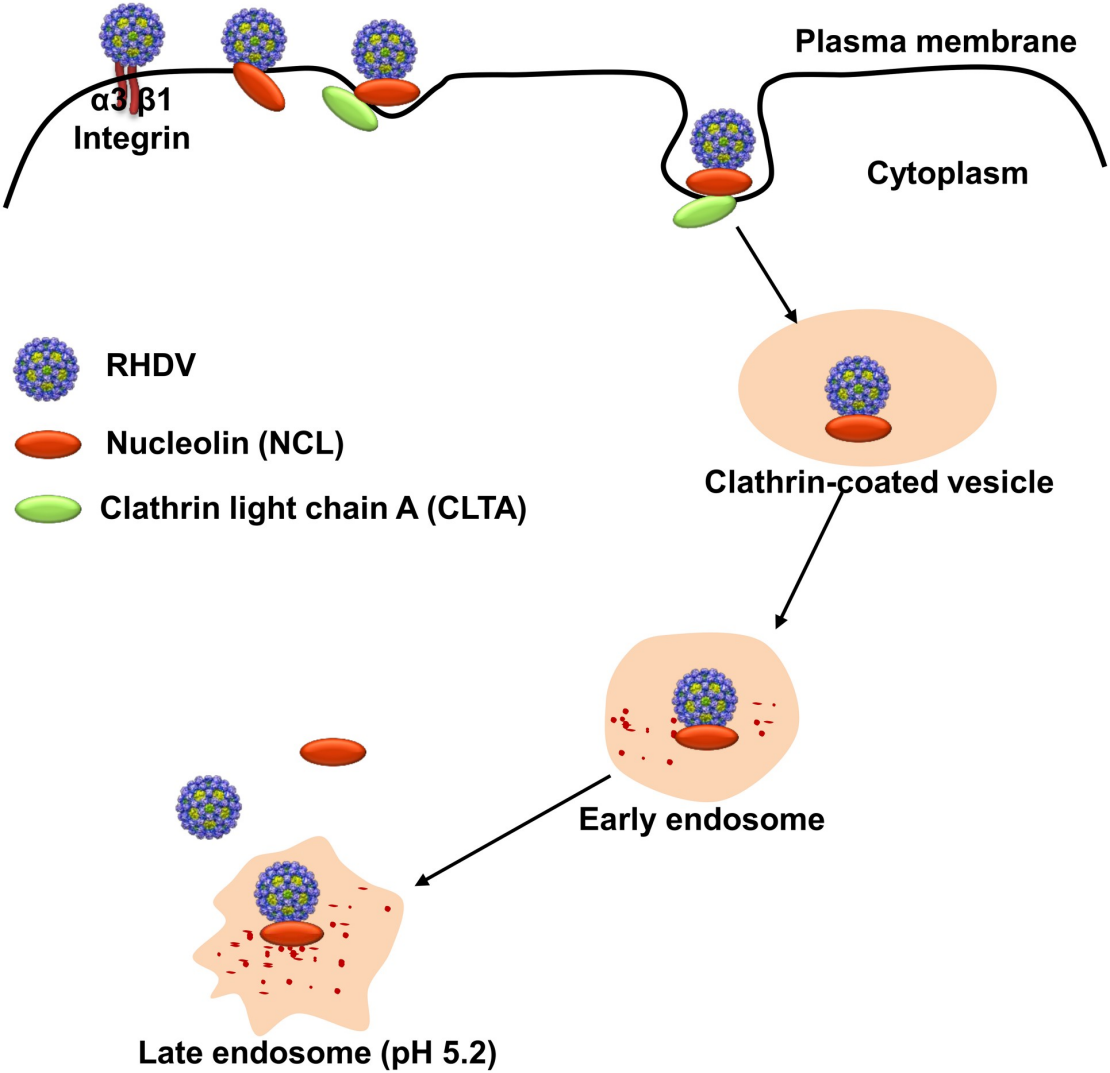
RHDV internalization model. First, RHDV attaches to the RK-13 cell surface by binding to specific cell receptors. Then, the capsid protein of RHDV and the host clathrin are bridged by NCL, and form a protein complex. Finally, RHDV is internalized by the host cell via clathrin-dependent endocytosis.

In conclusion, this study is the first to report that NCL plays a key role in the infection mechanism of RHDV. As shown in Fig 10, the capsid protein of RHDV and the host clathrin are connected through NCL and form a triprotein complex, thereby completing the intrusion process of RHDV through clathrin-dependent endocytosis. To our knowledge, this is the first report of the infection mechanism of RHDV at the molecular level. These results enrich current knowledge of the pathogenic mechanism of RHDV and provide important clues for the development of novel vaccines against RHDV infection.

## Materials and methods

### Ethics statement

All experiments were performed in a secondary biosecurity laboratory. All experiments involving rabbits and mice were carried out in strict accordance with the recommendations of the Guide for the Care and Use of Laboratory Animals of the Ministry of Science and Technology of the People’s Republic of China, and all efforts were made to minimize suffering. All animal procedures were approved by the Institutional Animal Care and Use Committee of the Shanghai Veterinary Research Institute, Chinese Academy of Agricultural Sciences (permit number: SHVRIAU-7-0103).

### Cell lines and viruses

Rabbit kidney cells (RK-13, ATCC CCL37) and 293T cells (ATCC, CRL-3216) were grown in minimal essential medium (Life Technologies, Carlsbad, CA, USA) or Dulbecco’s modified Eagle’s medium (DMEM) (Life Technologies) supplemented with 10% fetal bovine serum under an atmosphere of 5% CO_2_ at 37°C. RHDV strain JX/CHA/97 was isolated in 1997 during an outbreak of RHDV in China and stored in our laboratory. mRHDV was constructed in 2017 with a reverse genetics technique and stored in our laboratory [8].

### Plasmid constructs

The p3×FLAG-CMV-14 vector (Sigma-Aldrich Corporation, St. Louis, MO, USA) and pCMV-Myc (Clontech Laboratories, Inc., Mountain View, CA, USA) were used to create mammalian expression constructs. The pGEX-4T-1 vector (GE Healthcare Life Sciences, Chicago, IL, USA) was expressed in competent E. *coli* BL21-CodonPlus (DE3) cells. NCL (GenBank accession number XM_017343189.1) and CLTA (GenBank accession number XM_002707987.3) sequences were amplified by reverse transcription polymerase chain reaction (RT-PCR) from a RK-13 cell cDNA library. Total RNA was isolated from RK-13 cells using TRIzol reagent (Invitrogen Corporation, Carlsbad, CA, USA) according to the manufacturer’s instructions. DNA was removed from the isolated RNA using DNaseI (Takara Bio, Inc., Shiga, Japan) and cDNA was produced with Moloney murine leukemia virus reverse transcriptase (Promega Corporation, Madison, WI, USA) and random hexamers. The RHDV *VP60* gene was amplified by RT-PCR from RHDV cDNA. The genomic sequence of RHDV CHA/JX/97 was retrieved from the GenBank database (accession number DQ205345). Viral cDNA was generated as described in our previous report [47]. All plasmids were created with In-Fusion HD Cloning Kits (Clontech Laboratories, Inc.) according to the manufacturer’s instructions. All RT-PCR amplifications for cloning were performed with TransStart FastPfu Fly DNA Polymerase (TransGen Biotech Co., Ltd., Beijing, China) according to the manufacturer’s instructions. RT-PCR products were separated by agarose gel electrophoresis and purified with the SanPrep Column DNA Gel Extraction Kit (Sangon Biotech Co., Ltd., Shanghai, China). Restriction digests were performed using commercial kits (New England Biolabs, Ipswich, MA, USA) according to the manufacturer’s instructions. All plasmid sequences were amplified by RT-PCR and analyzed by Sanger sequencing to verify the sequence fidelity and reading frames (Sangon Biotech Co., Ltd.). Details of all constructs used in the study, including residue numbers, expression vectors, and tags, are summarized in S1 Table. In addition, the primers used in this research are listed in S2 Table.

### Bacterial expression of recombinant proteins and purification

All proteins were expressed in competent E. *coli* BL21-CodonPlus (DE3) cells (TransGen Biotech Co., Ltd.) that were seeded in 1 mL of an overnight starter culture and then grown in 100 mL of lysogeny broth at 220 rpm and 37°C until the mid-log phase (OD_600_ = ~0.6–0.8). Then, the cells were typically induced with 0.2 mM isopropyl β-D-1-thiogalactopyranoside and incubated for approximately 16 h at 16°C and 220 rpm. Cells were pelleted by centrifugation at ~5,000 g and stored at -80°C. Bacterial pellets were resuspended in lysis buffer (20 mM Tris/HCl pH 7.4, 60 mM NaCl, 1 mM ethylenediaminetetraacetic acid, 1 mg/mL lysozyme, 1 mM dithiothreitol, and 0.1% Triton X-100) supplemented with complete protease inhibitor cocktail (Thermo Fisher Scientific, Waltham, MA, USA) for 1 h on ice. Nuclease was then added and the lysate was incubated for 1 h at ambient temperature under rotation. The lysates were centrifuged at 4°C for 10 min at 12,000 × g. Glutathione-Sepharose 4B beads (Pierce Biotechnology, Waltham, MA, USA) were added to the clarified supernatants and the mixtures were incubated overnight at 4°C under rotation. The beads were washed with lysis buffer, followed by three washes with phosphate-buffered saline (PBS), and then stored at 4°C in an equal volume of PBS.

### Glutathione-S-transferase (GST) pulldown assay

For the *in vitro* binding assay, Flag- or myc-tagged NCL, CLTA, and VP60 proteins were expressed in RK-13 cells. In accordance with the manufacturer’s instructions, the GST pull-down assay was performed by incubating 50 μL of a 50% slurry of glutathione-sepharose beads containing 25 μM GST fusion protein in lysis buffer with a three-fold molar excess of prey protein (Pierce Biotechnology). The bound proteins were separated by sodium dodecyl sulfate-polyacrylamide gel electrophoresis (SDS-PAGE) and then subjected to western blot analysis.

### Co-immunoprecipitation (Co-IP) analysis

RK-13 cells were co-transfected with the bait and prey plasmids. At 48 h after transfection, total protein was isolated from RK-13 cells using IP lysis buffer. Co-IP analysis was conducted using a commercial Co-IP kit (Pierce Biotechnology) according to the manufacturer’s instructions. AminoLink Plus Coupling Resin was incubated with anti-NCL monoclonal antibody (mAb) (Santa Cruz Biotechnology, Inc., Dallas, TX, USA) or anti-VP60 mAb, which was prepared in our laboratory, and then subjected to SDS-PAGE. Immunoblot analysis of the proteins was subsequently conducted using mAbs against NCL, VP60, CLTA, and AP2 (Abcam, Cambridge, UK).

### Western blot analysis

Protein samples were separated on 12% gels and then transferred to nitrocellulose membranes (Hybond-C; Amersham Life Sciences, Little Chalfont, UK) using a semi-dry transfer apparatus (Bio-Rad Laboratories, Hercules, CA, USA). The membranes were blocked with 5% (w/v) nonfat milk in TBST buffer (150 mM NaCl, 20 mM Tris, and 0.1% Tween-20, pH 7.6) for 3 h at 4°C and then stained overnight at 4°C with a primary antibody (Ab). After washing three times for 10 min each time, the membranes were incubated with a secondary Ab against immunoglobulin G (IgG) conjugated to horseradish peroxidase (Sigma-Aldrich Corporation) in PBST buffer (137 mM NaCl, 2.7 mM KCl, 10 mM Na_2_HPO_4_, 2 mM KH_2_PO_4_, and 0.1% Tween-20 pH 7.4) for 1 h at room temperature (RT). Finally, after washing three times for 10 min each time, the proteins were detected using an automatic chemiluminescence imaging analysis system (Tanon Science & Technology Co., Ltd., Shanghai, China).

### Immunofluorescence assay (IFA)

Cells were fixed in 3.7% paraformaldehyde in PBS (pH 7.5) at RT for 30 min and subsequently permeabilized by incubation in methanol at -20°C for 30 min. The fixed cells were blocked with 5% (w/v) nonfat milk in PBST buffer for 3 h at 4°C and then stained with a primary Ab for 2 h at 37°C. After washing three times for 10 min each time, the cells were incubated with a secondary Ab against IgG conjugated to fluorescein isothiocyanate (FITC) (Sigma-Aldrich Corporation) in PBST buffer for 1 h at RT. Finally, after washing three times for 10 min each time the samples were observed under a fluorescence microscope equipped with a video documentation system (Nikon Corporation, Tokyo, Japan).

### Attachment and internalization of RHDV

The kinetics of RHDV attachment was studied by adding the virus at a MOI of 0.25–2 to chilled RK-13 cells. After incubation for 2 h at 4°C, the cells were washed extensively with chilled PBS and then lysed. For the internalization analysis, the cells were washed extensively with chilled PBS following attachment for 2 h and the temperature was shifted to 37°C to allow internalization of the attached viruses. At specific time points post-internalization, the cells were washed extensively with acidic PBS (pH 2.5) and PBS to remove viruses attached to the cell surface. The washed cells were lysed and the total RNA of each lysate sample was extracted with the Spin Column RNA Cleanup & Concentration Kit (Sangon Biotech Co., Ltd.).

### Fluorescent ligand uptake and measurement

NCL siRNA-treated (24 h post-transfection) RK-13 cells were serum-starved in DMEM for 3 h, rinsed extensively with chilled PBS, and cold-bound with Alexa Fluor 488-conjugated epidermal growth factor (EGF), Alexa Fluor 488-conjugated transferrin, Alexa Fluor 488-conjugated dextran, and Alexa Fluor 488-conjugated CD4 at concentrations of 2 μg/mL for 1 h at 4°C, as recommended by the manufacturer (Life Technologies). Unbound ligands were removed by extensive washing with cold PBS and the cells were incubated in warm DMEM at 37°C for the designated times. At the indicated time-points, non-internalized ligands were removed by washing with acidic PBS (pH 2.5). Internalized fluorescent signals in the cells were measured by flow cytometry.

To image the internalization of single RHDV particles in live cells, purified virions were conjugated to the fluorescent dye Alexa Fluor 488 (Molecular Probes, Invitrogen Corporation), solubilized in dimethyl sulfoxide (DMSO) at 10 mg/mL, and incubated at a final concentration of 50 μg/mL with purified RHDV (1 mg/mL) in 0.1 M NaHCO_3_ (pH 8.3) for 90 min at RT. The viruses were separated from free dye by differential centrifugation, solubilized in PBS containing 10 mM HEPES (4-(2-hydroxyethyl)-1-piperazineethanesulfonic acid; pH 7.4), and stored at -80°C. To measure the effect of labeling on viral internalization in RK-13 cells, equivalent amounts of total viral protein from labeled and unlabeled preparations were titrated by quantitative real-time polymerase chain reaction (qRT-PCR).

### Effects of inhibitors on RHDV internalization

RK-13 cells were pretreated for 30 min at 37°C with each of the inhibitors followed by cold-synchronized infection. Where indicated, inhibitors were added to previously infected cells at 2 or 3 hpi. Stock solutions of 50 mM nystatin (Nys) (Sigma-Aldrich Corporation) and 50 mM 5-ethylisopropyl amiloride (EIPA) (Sigma-Aldrich Corporation) were dissolved in DMSO. Water was used as a solvent for 10 mM chlorpromazine (CPZ) (Sigma-Aldrich Corporation). All inhibitors were present throughout the experiments. This study was required given that we used the highest doses reported in the literature to ensure the efficacy of the inhibitors. We also assessed the cytotoxic activity of the organic solvent DMSO. Based on the results of these experiments, optimal non-toxic working concentrations were selected for the internalization assay. For the blocking experiments, RK-13 cells were pretreated in serum-free minimal essential medium containing DMSO as a control, 100 mM Nys, 50 mM EIPA, or 30 mM CPZ. These drug-treated cells were subsequently infected with RHDV at a MOI of 1, incubated in growth medium for 1 h at 37°C, and harvested for flow cytometry or qRT-PCR analysis.

### qRT-PCR analysis

The vRNA-VP60 copy numbers of attached and internalized mRHDV were estimated by qRT-PCR. The amount of mRNA in each sample was normalized to that of rabbit glyceraldehyde 3-phosphate dehydrogenase (GADPH). Total RNA was extracted from cell lysate using TRIzol reagent (Invitrogen Corporation) according to the manufacturer’s instructions. All qRT-PCR reactions were performed with the SYBR PreMix Ex Taq II kit (Takara Bio, Inc.) with an ABI 7500 Fast Real Time PCR system (Applied Biosystems, Carlsbad, CA, USA). For VP60, the qRT-PCR reaction was performed as follows: one cycle at 95°C for 30 s, then 40 cycles at 95°C for 5 s, 58°C for 5 s and 72°C for 30 s, and one cycle to generate a melting curve. The primers are listed in S2 Table.

### Immunization and viral challenge

Twenty 8-week-old New Zealand male rabbits seronegative for RHDV were randomly distributed into four groups (n = 5/group) and housed in individual ventilated cages. All experimental protocols were reviewed by a state ethics commission and have been approved by the competent authority. The details of the protection assay are shown in S4 Table. Rabbits were immunized with DVN peptide (1 mg), a control peptide (1 mg), a commercial inactivated RHDV vaccine (Nanjing Tianbang Bio-industry Co., Ltd., Nanjing, China), or PBS, respectively. All of the groups were immunized on day 0. Blood samples were collected from the marginal ear vein before each immunization and 2 weeks after the last injection to analyze the level and specificity of the Ab response against RHDV. Ab titers were assessed using an enzyme-linked immunosorbent assay (ELISA). Briefly, RHDV strain JX/CHA/97 (100 μL, 1 μg/mL, incubated overnight at 4°C) was used to capture Abs in the sera (incubated for 1 h at 37 °C) and then detected with 100 μL of horseradish peroxidase-conjugated mouse anti-rabbit IgG (Sigma-Aldrich Corporation) per well (diluted 1:5000 in PBS containing 0.5% Tween 20 and 10% fetal bovine serum), followed by 100 μL of tetramethylbenzidine liquid substrate (Sigma-Aldrich Corporation) per well for 30 min at RT in the dark. End-point titers were defined as the highest plasma dilution that resulted in an absorbance value (OD_450_). Data are presented as log10 values. In addition, all rabbits were challenged intramuscularly with 100 × the median lethal dose (LD_50_) of RHDV at 21 days after immunization. The rabbits were clinically examined daily for 7 days post-challenge. All animals were necropsied and serum and organs were collected and stored at -30°C. For histopathological analysis, samples were fixed in 10% neutral buffered formalin solution, sectioned, and stained with hematoxylin and eosin. Alternatively, immunohistochemical analysis was conducted with a VP60-specific mAb. To evaluate the efficiency of the cellular responses, the serum levels of interferon (IFN)-γ, interleukin (IL)-2 and IL-4 at weeks 0, 1, and 2 post-immunization were measured using commercially available ELISA kits (R&D Systems, Inc., Minneapolis, MN, USA) according to the manufacturer’s instructions.

### Mass spectrometry

Jingjie PTM BioLab Co., Ltd. (Hangzhou, China) performed all mass spectrometry analyses.

### Statistical analyses

Statistical analyses were conducted with the Student’s *t*-test and one-way analysis of variance using SAS 9.1 software (SAS Institute, Cary, NC, USA). Probability (*p*) values of < 0.05 and < 0.01 were considered as significant and extremely significant, respectively.

## Supporting information

S1 Fig NCL siRNA knockdown of NCL at the RK-13 cell surface. The expression level of NCL was determined by western blot analysis with anti-NCL mAb. RK-13 cells were transfected with NCL siRNA (100 nM) or non-specific siRNA (100 nM) for 24 h and then lysed. β-actin was employed as an internal control.
(TIF)

S2 Fig NCL-Flag and Myc-CLTA expression in RK-13 cells. The expression levels of NCL **(A)** or CLTA **(B)** were determined by western blot analysis with anti-Flag mAb or anti-Myc mAb. RK-13 cells were transfected with pNCL-Flag (2 μg) or pMyc-CLTA-Flag (2 μg) for 48 h and then lysed. The pCMV-Myc vector and p3×FLAG-CMV-14 vector acted as negative controls. β-actin was employed as an internal control.
(TIF)

S3 Fig Kinetics of mRHDV entry into RK-13 cells. **(A)** Kinetics of mRHDV attachment to RK-13 cells. Increasing amounts of mRHDV were added (0.25–2 MOI) to chilled RK-13 cells. After 2 h of attachment, the cells were washed and lysed. The amount of attached virus was determined by qRT-PCR and expressed as VP60 RNA copies per 100,000 copies of GADPH. **(B)** Kinetics of mRHDV internalization into RK-13 cells. Internalization of mRHDV (MOI = 1) into RK-13 cells was assessed by mRHDV incubation for 10, 30, and 60 min at 37°C. The amount of internalized virus was determined by qRT-PCR and expressed as VP60 RNA copies per 100,000 of GADPH.
(TIF)

S4 Fig NCL is involved in clathrin-dependent endocytosis of RK-13 cell. Uptake of EGF-Alexa 488 **(A)**, transferrin-Alexa 488 **(B)**, dextran-Alexa 488 **(C)** or CD4-Alexa 488 **(D)** by RK-13 cells treated with NCL siRNA or non-specific siRNA, as quantified by flow cytometry.
(TIF)

S1 Table Plasmid construct details. (XLSX)

S2 Table Oligonucleotide primer sequences. (XLSX)

S3 Table Details of protein expression. (XLSX)

S4 Table Details of the protection assay after challenging with virulent RHDV. (DOCX)
