# Supplementary figures and images for: Nucleolin mediates the internalization of rabbit hemorrhagic disease virus through clathrin-dependent endocytosis
Source: PLoS Pathog. 2018 Oct 19;14(10):e1007383. doi: 10.1371/journal.ppat.1007383 (PMC6209375; doi:10.1371/journal.ppat.1007383)

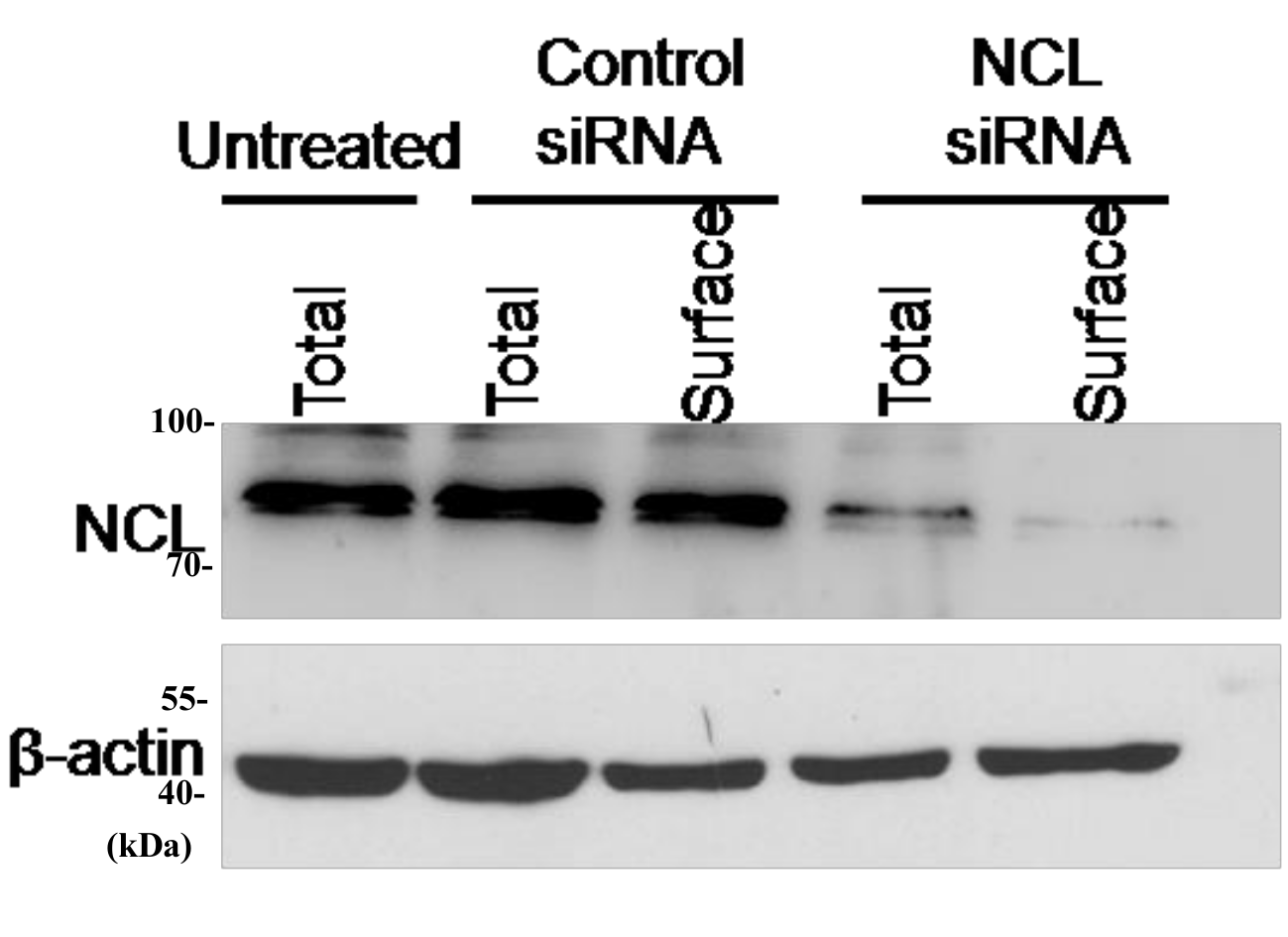

Supplement: S1 Fig — The expression level of NCL was determined by western blot analysis with anti-NCL mAb. RK-13 cells were transfected with NCL siRNA (100 nM) or non-specific siRNA (100 nM) for 24 h and then lysed. β-actin was employed as an internal control. (TIF) [file ppat.1007383.s001.tif]

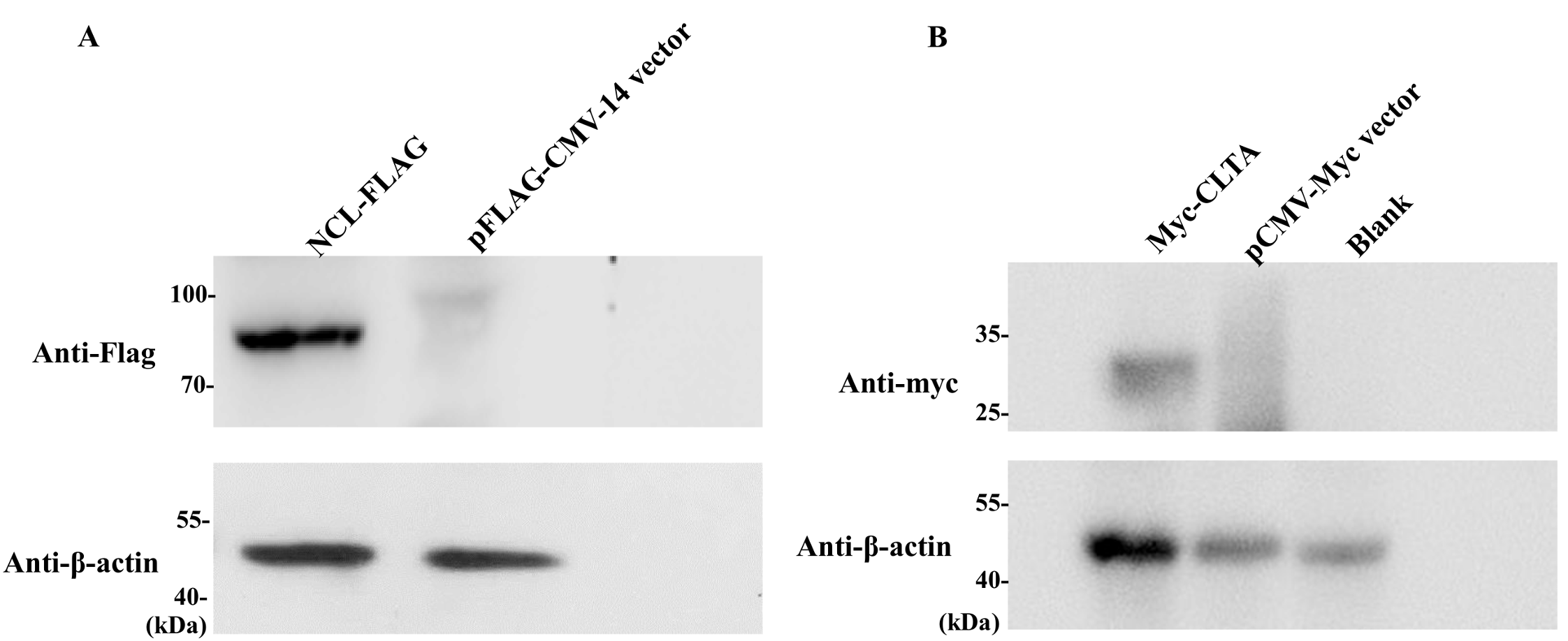

Supplement: S2 Fig — The expression levels of NCL (A) or CLTA (B) were determined by western blot analysis with anti-Flag mAb or anti-Myc mAb. RK-13 cells were transfected with pNCL-Flag (2 μg) or pMyc-CLTA-Flag (2 μg) for 48 h and then lysed. The pCMV-Myc vector and p3×FLAG-CMV-14 vector acted as negative controls. β-actin was employed as an internal control. (TIF) [file ppat.1007383.s002.tif]

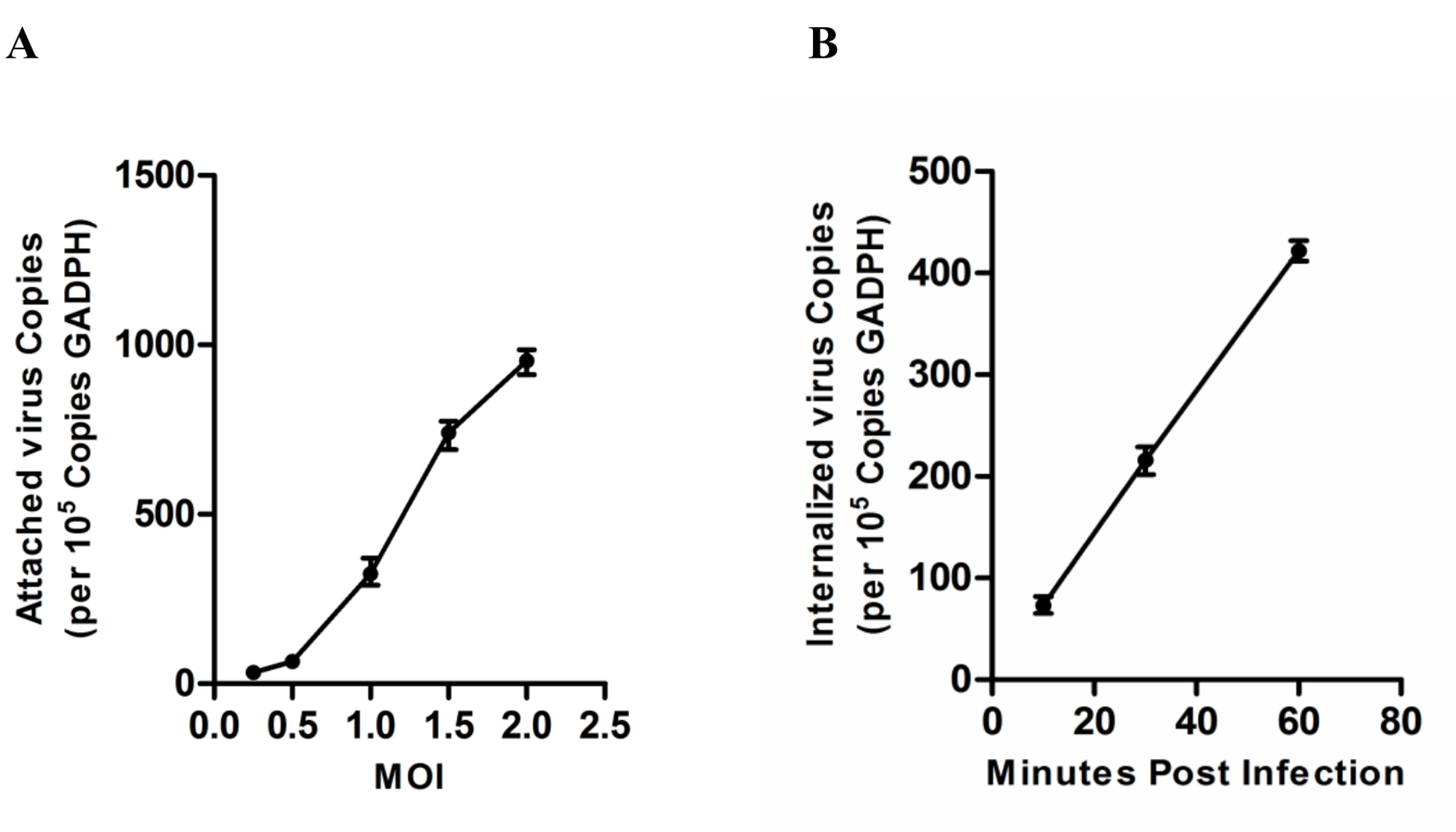

Supplement: S3 Fig — (A) Kinetics of mRHDV attachment to RK-13 cells. Increasing amounts of mRHDV were added (0.25–2 MOI) to chilled RK-13 cells. After 2 h of attachment, the cells were washed and lysed. The amount of attached virus was determined by qRT-PCR and expressed as VP60 RNA copies per 100,000 copies of GADPH. (B) Kinetics of mRHDV internalization into RK-13 cells. Internalization of mRHDV (MOI = 1) into RK-13 cells was assessed by mRHDV incubation for 10, 30, and 60 min at 37°C. The amount of internalized virus was determined by qRT-PCR and expressed as VP60 RNA copies per 100,000 of GADPH. (TIF) [file ppat.1007383.s003.tif]

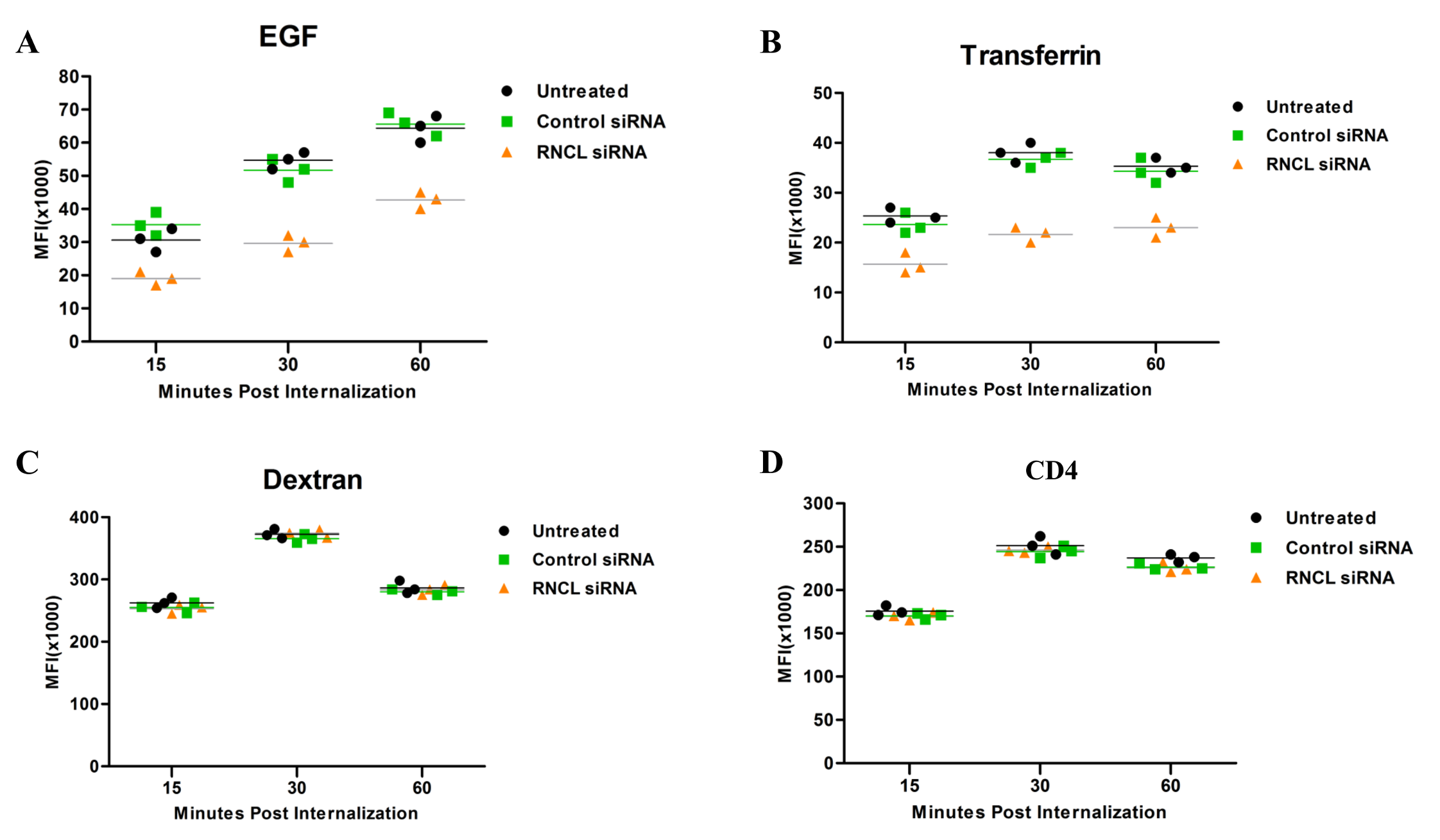

Supplement: S4 Fig — Uptake of EGF-Alexa 488 (A), transferrin-Alexa 488 (B), dextran-Alexa 488 (C) or CD4-Alexa 488 (D) by RK-13 cells treated with NCL siRNA or non-specific siRNA, as quantified by flow cytometry. (TIF) [file ppat.1007383.s004.tif]
