# Supplementary material for: Nucleolin mediates the internalization of rabbit hemorrhagic disease virus through clathrin-dependent endocytosis
Source: PLoS Pathog. 2018 Oct 19;14(10):e1007383. doi: 10.1371/journal.ppat.1007383 (PMC6209375; doi:10.1371/journal.ppat.1007383)
Supplement: S4 Table — (DOCX) [file ppat.1007383.s008.docx]

**S4 Table. The details of protection assay after challenging with virulent RHDV**

| Group | Number | Antigen | Dosage | Method of immunization | Challenge experiment | | | |
| --- | --- | --- | --- | --- | --- | --- | --- | --- |
|  |  |  |  |  | Pathogen | Method  of infection | Clinical signs | Protection level |
| I | 5 | DVN peptide^a^ | 1 mg | Subcutaneous | RHDV (JX/CHA/97) | Intramuscular  injection | − − − + + | 3/5(60%) |
| III | 5 | Control peptide^b^ | 1 mg |  |  |  | + + + + + | 0/5(0%) |
| IV | 5 | Commercial vaccine | 1 dose |  |  |  | − − − − + | 4/5(80%) |
| V | 5 | PBS | 2 mL |  |  |  | + + + + + | 0/5(0%) |

^a^ The amino acid sequence for DVN peptide is RRTGDVNAAAGSTNGTQ.

^b^ The amino acid sequence for control peptide is RHDV VP60 residues 434-450 region: VTYTPQPDRIVTTPGTP.
